# In vivo topical gene therapy for recessive dystrophic epidermolysis bullosa: a phase 1 and 2 trial

**DOI:** 10.1038/s41591-022-01737-y

**Published:** 2022-03-28

**Authors:** Irina Gurevich, Pooja Agarwal, PeiPei Zhang, John A. Dolorito, Stacie Oliver, Henry Liu, Nicholas Reitze, Nikhil Sarma, Isin Sinem Bagci, Kunju Sridhar, Visesha Kakarla, Vamsi K. Yenamandra, Mark O’Malley, Marco Prisco, Sara F. Tufa, Douglas R. Keene, Andrew P. South, Suma M. Krishnan, M. Peter Marinkovich

**Affiliations:** 1 Program in Epithelial Biology and Department of Dermatology, Stanford University School of Medicine, Stanford, CA USA; 2 Krystal Biotech, Pittsburgh, PA USA; 3 Sidney Kimmel Medical College, Thomas Jefferson University, Philadelphia, PA USA; 4 Microscopy Unit, Shriners Hospital for Children, Portland, OR USA; 5 Veterans Affairs Medical Center, Palo Alto, Stanford, CA USA

**Keywords:** Genetics research, Translational research

## Abstract

Recessive dystrophic epidermolysis bullosa (RDEB) is a lifelong genodermatosis associated with blistering, wounding, and scarring caused by mutations in *COL7A1*, the gene encoding the anchoring fibril component, collagen VII (C7). Here, we evaluated beremagene geperpavec (B-VEC), an engineered, non-replicating *COL7A1* containing herpes simplex virus type 1 (HSV-1) vector, to treat RDEB skin. B-VEC restored C7 expression in RDEB keratinocytes, fibroblasts, RDEB mice and human RDEB xenografts. Subsequently, a randomized, placebo-controlled, phase 1 and 2 clinical trial (NCT03536143) evaluated matched wounds from nine RDEB patients receiving topical B-VEC or placebo repeatedly over 12 weeks. No grade 2 or above B-VEC-related adverse events or vector shedding or tissue-bound skin immunoreactants were noted. HSV-1 and C7 antibodies sometimes presented at baseline or increased after B-VEC treatment without an apparent impact on safety or efficacy. Primary and secondary objectives of C7 expression, anchoring fibril assembly, wound surface area reduction, duration of wound closure, and time to wound closure following B-VEC treatment were met. A patient-reported pain–severity secondary outcome was not assessed given the small proportion of wounds treated. A global assessment secondary endpoint was not pursued due to redundancy with regard to other endpoints. These studies show that B-VEC is an easily administered, safely tolerated, topical molecular corrective therapy promoting wound healing in patients with RDEB.

## Abstract (web summary)

Preclinical data and results from a phase 1 and 2 trial demonstrate preliminary safety and efficacy of topical gene therapy for recessive dystrophic epidermolysis bullosa

## Main

Recessive dystrophic epidermolysis bullosa (RDEB) is a devastating rare genetic skin disease with a prevalence of 1.35 per million in the United States^1^. RDEB is caused by mutations in *COL7A1*, the gene encoding collagen VII (C7), which is assembled into anchoring fibrils (AFs), the basement membrane structures that anchor the epidermis and dermis together. C7 contains two essential non-collagenous (NC) domains: NC1, which inserts AFs into the basement membrane^2,3^, and NC2, which promotes AF assembly^4,5^. C7 defects impair dermal–epidermal cohesion and produce lifelong widespread painful blistering and fibrosis starting at birth, accompanied by scarring, susceptibility to infection and a predisposition to skin cancer^5,6^. This disease is extremely burdensome to patients^7^, however, no approved corrective therapies for RDEB currently exist.

A number of earlier C7 replacement strategies have been evaluated for RDEB^8^. The first approach, bone marrow transplantation, promoted C7 expression and wound healing; however, long-term results remain unclear and the procedure is associated with a mortality rate approaching 30% ^9^. Another earlier approach, graft placement of ex vivo *COL7A1* retrovirally modified autologous keratinocytes, promoted C7 expression and durable wound healing in RDEB skin, however, this varied from patient to patient and slowly declined over time^10,11^. A similar autologous keratinocyte ex vivo approach was used to promote laminin-332 expression and wound healing in junctional epidermolysis bullosa skin using a *LAMB3* retroviral vector^12^. Grafts in these studies required general anesthesia, a specialized surgical team for graft placement, and postoperative graft immobilization procedures ranging from a 1 week hospitalization for *COL7A1* RDEB grafts^11^ to induction of prolonged coma in an isolation chamber for *LAMB3* junctional epidermolysis bullosa grafts^12^. Transfer of ex vivo lentiviral-modified *COL7A1*-expressing RDEB fibroblasts to the skin of patients with RDEB has also been studied^13^. This approach does not require skin grafting and instead injects genetically modified fibroblasts directly into the skin. However, as in the other ex vivo cell engineering approaches, this technique still requires collection of skin tissue followed by resource-intensive cell engineering and expansion at specialized manufacturing centers^11–13^.

Correction of genetic skin diseases via direct gene transfer in vivo has been a longstanding yet unrealized goal in the gene therapy field. Previous attempts at naked DNA skin transfer have proven insufficient for disease correction^14^. Although viral vectors augment in vivo gene transfer efficiency, undesired immune reactions often result, especially upon repeat treatment^15^. Herpes simplex virus type 1 (HSV-1) vectors, in contrast, both efficiently infect cells^16^ and resist immune clearance^17^, which is partially explained by the innate immune-evasive properties of HSV tegument proteins^18^ and evidence suggesting that HSV-1 infected cell protein 47 (ICP47) directly inhibits the transporter associated with antigen processing^19^. Natural immune-evading functions coupled with deletion of pro-inflammatory genes from the HSV-1 vector backbone^20,21^ make newer generation modified HSV-1 vectors especially well-suited for gene therapy.

The large ~9 kb *COL7A1* transgene needed for RDEB skin correction poses additional challenges for vector engineering. Beyond the capacity of most viral vectors, including adenoviruses and adeno-associated viruses, *COL7A1* can only be functionally expressed in lentiviral and retroviral vectors after extensive vector modifications^11,13^. HSV-1 viruses, in contrast, have transgene payload capacities exceeding 30 kb. Because they are non-integrating and episomal, HSV-1 vectors do not pose any insertional mutagenesis risk. In total, these properties make HSV-1 vectors particularly suitable for in vivo direct gene transfer.

We describe here the development and clinical translation of a topical gene therapy treatment for RDEB that can be repeatedly applied without serious adverse events. Beremagene geperpavec (B-VEC), a replication-defective HSV-1 vector containing two copies of the *COL7A1* coding sequence, efficiently restored C7 expression in RDEB keratinocytes and fibroblasts in vitro. Topical B-VEC promoted skin integrity and robust C7 expression, followed by its assembly into basement membrane-associated AFs in vivo in C7-deficient mice and primary human RDEB skin xenografts. With preclinical data providing the scientific rationale, we evaluated the clinical translation to humans in a phase 1 and 2 exploratory study, that is, the first-ever clinical trial of topical gene therapy. Taken together, we demonstrate here a novel, easy-to-administer, and highly accessible gene therapy capable of reversing genetic disease through repeated application directly to patient skin wounds.

## Results

### Restoration of C7 expression in RDEB patient cell culture

**Fig. 1:**
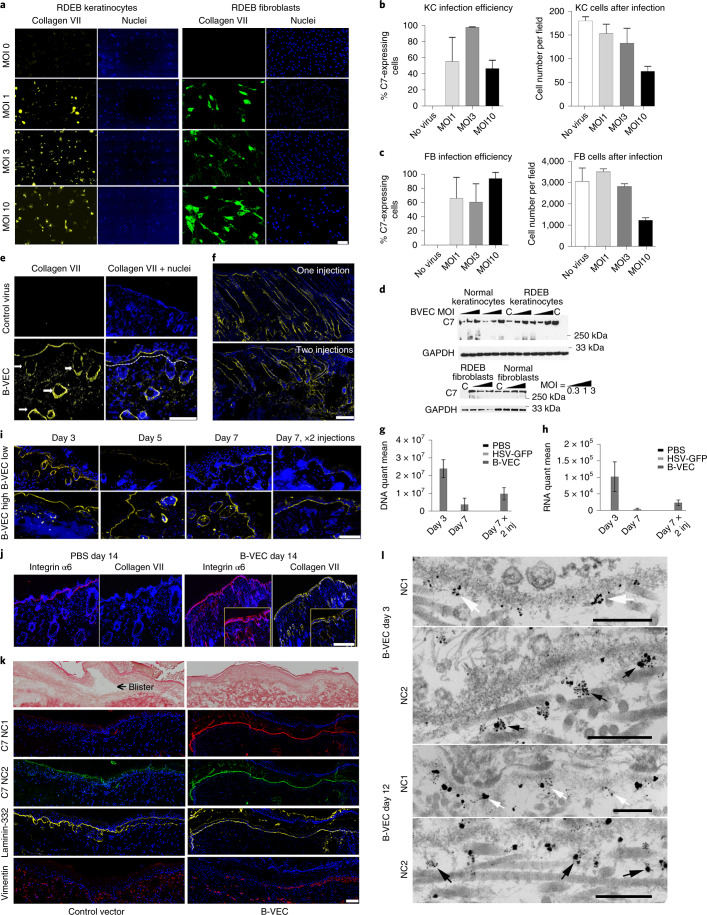
Collagen VII (C7) expression in primary RDEB patient cells, RDEB mice, and human RDEB xenografts on immunodeficient mice following B-VEC therapy. **a**, RDEB keratinocytes and fibroblasts were infected in vitro with B-VEC vector at various ratios of cell to viral particle (MOI). Cells were fixed 48 h after infection and analyzed by indirect immunofluorescence microscopy (IDIF) to validate C7 expression in keratinocytes (kc; yellow) and fibroblasts (fb; green). Scale bar, 100 µm. **b**,**c**, Infection efficiency and live cell number in cultures after infection (*n* = 3 for each condition). **d**, Keratinocytes and fibroblasts were collected after various MOI (*n* = 3 for each condition) and analyzed by Western blot. C, control vector. **e**, Mice were injected intradermally at four separate sites on the back (one vehicle control site and three B-VEC treatment sites, one injection per site). Some mice received a second injection on day 3 at the same four sites. At day 3 after B-VEC treatment (dose of 4.6 x 10^7^ p.f.u. per 50 µl per injection), RDEB mice injected intradermally with B-VEC vector had linear C7 basement membrane zone (BMZ) expression (yellow, dotted line) including in hair follicle basement membranes (arrows). Scale bar, 100 µm. f, At day 7, C7 expression (yellow) was analyzed using IDIF after one or two injections in RDEB mouse skin. Scale bar, 200 µm. **g**,**h**, *COL7A1* DNA (**g**) and C7 transcript expression levels (**h**) were analyzed using qPCR or RT–qPCR, after one or two injections (dose of 4.6 x 10^7^ p.f.u. per 50 µl per injection) in RDEB mouse skin, on day 3 and 7 after single or repeated injections (*n* = 9 injections, triplicate for each condition, per timepoint). **i**, Demonstration of C7 expression dose dependency in RDEB mouse skin for low-dose injections (top row) and high-dose injections (bottom row) at days 3, 5, and 7 after injection. The far right panels show C7 expression at high and low B-VEC doses 7 days after both injections. **j**, Heterozygous RDEB mice were treated with daily topical B-VEC or vehicle (PBS) applications on wounded skin for 5 days. Fourteen days after the first application, linear C7 expression using human-specific antibody is shown in yellow at the epidermal–dermal junction and hair follicle epithelial–dermal junctions, and co-localization with the BMZ marker α6 integrin staining is shown in red. The inserts in the right panels demonstrate higher magnification of the sample region. Four areas on the back were treated per mouse (1 control and 3 B-VEC) and four mice in total were tested. Scale bar, 100 µm. **k**, Xenografts comprising human RDEB fibroblasts and keratinocytes were treated by topical B-VEC and imaged 5 days later. The grafts were analyzed by light (first row) and immunofluorescence (rows 2–5) microscopy. Light microscopy demonstrated areas of dermal–epidermal blistering (left panel, arrow), which was not seen after B-VEC treatment (right panel). In the treated region, expression of the NC1 (red) and NC2 (green) domains of C7 co-localized with the control BMZ marker laminin-332 (yellow). Row 5 demonstrates the persistence and location of human fibroblasts in the dermis of the graft. Scale bar, 100 µm. Representative of eight grafts treated with B-VEC (mice) and 2 with placebo. **l**, Human RDEB skin xenografts were treated with topical B-VEC and analyzed after 3 and 12 days using immunoelectron microscopy with C7 NC1-directed antibodies (NP185), followed by gold nanoparticles (first, third rows; white arrows), and NC2-directed primary antibodies (LH24), followed by gold nanoparticles (second, fourth rows; black arrows). Error bars on all panels represent standard error of the mean of all replicates. Representative of eight grafts treated with B-VEC and two grafts treated with placebo. Scale bars, 300 nm. Data plots, including error bars and *P* values, were generated using GraphPad Prism v8.3.0.

**Extended Data Fig. 1:**
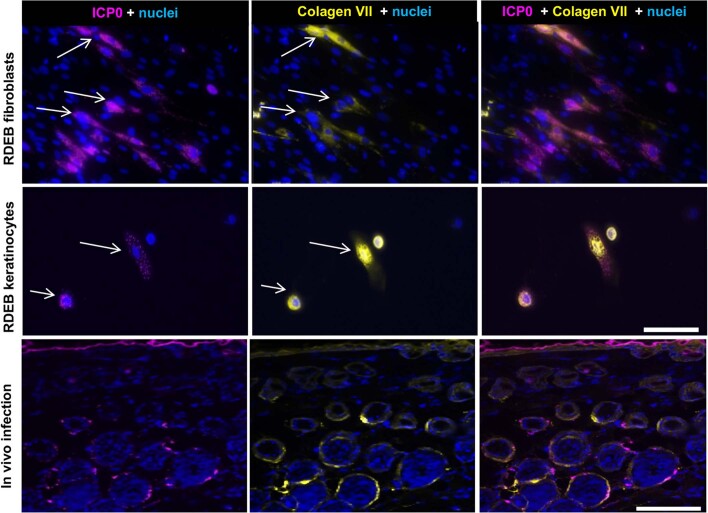
Co-expression of C7 with B-VEC viral markers. RDEB fibroblasts and keratinocytes were infected in vitro with B-VEC vector (top 2 rows). Representative of 3 replicates for each condition. RDEB mice were injected intradermally with B-VEC vector (bottom row). Representative of 3 mice; 3 treated back areas per mouse. Co-expression of C7 (yellow) and ICP0 (pink) is shown in RDEB fibroblast culture (top panel), keratinocyte culture (middle panel), and in mouse skin (bottom panel). Scale bar is 100 μm. B-VEC, beremagene geperpavec; RDEB, recessive dystrophic epidermolysis bullosa.

To initially evaluate B-VEC as a cutaneous gene delivery vector, we examined its ability to promote C7 expression in primary skin cells of patients with RDEB in vitro. As seen in Fig. 1a, C7-null primary RDEB patient keratinocyte and fibroblast cultures demonstrated C7 expression 48 hours after B-VEC treatment. Dose-dependent increases in transduction efficiency were also demonstrated, targeting up to 100% of cells at a multiplicity of infection (MOI) of 1, 3 and 10 (Fig. 1b,c), with a slowing of proliferation observed at an MOI of 10 after 48 hours. Western blot analysis of primary RDEB keratinocyte and fibroblast cell lysates identified a dose-dependent increase in the expression of full-length C7 (Fig. 1d). Demonstrating that C7 expression is attributable to B-VEC, the expression of the HSV-1 early protein, ICP0, closely correlated with C7 expression in vitro (Extended Data Fig. 1). These data demonstrate that B-VEC is capable of gene delivery and expression in the specific C7-contributing cell types in patient skin.

### Molecular correction of C7 expression in vivo

To evaluate in vivo B-VEC-mediated *COL7A1* gene delivery, intradermal B-VEC injections were administrated to C7-deficient mice, which closely recapitulate the RDEB skin phenotype^22^. We performed three sets of experiments with the RDEB mice cohort, using three mice per cohort. Each mouse received three injections of B-VEC and one saline injection (*n* = 27 B-VEC, *n* = 9 saline). Some mice received a second injection on day 3 at the same four sites. At day 3 after B-VEC treatment (dose of 4.6 × 10^7^ p.f.u. per 50 µl per injection), C7 protein was localized in a linear pattern (Fig. 1e) at the dermal–epithelial junctions of the epidermis and in hair follicles. At day 7, widespread continuous linear C7 distribution through a larger area of skin was detected (as shown by joining multiple overlapping microscopy fields by tile imaging; Fig. 1f). *COL7A1* transgene delivery and expression was detected using quantitative polymerase chain reaction with or without reverse transcription (RT–qPCR or qPCR) at two time points, at 3 and 7 days, after a single or two injections (Fig. 1g,h). Evaluation of C7 expression kinetics (day 3, day 5, day 7) using indirect immunofluorescence microscopy (IDIF) demonstrated C7 expression in treated skin at both high doses (4.6 × 10^7^ p.f.u. per 50 µl per injection) and low doses (4.6 × 10^6^ p.f.u. per 50 µl per injection) of the virus (Fig. 1i). Because homozygous RDEB mice are too fragile to tolerate a wounding and topical application assessment, heterozygous RDEB mouse skin was evaluated following topical application of B-VEC on wounded skin. Linear C7 expression, as detected using human-specific C7 antibodies, was observed 14 days after treatment (Fig. 1j).

To further evaluate the in vivo effect of B-VEC, we studied primary regenerated human C7-null RDEB skin xenografted onto immunodeficient mice, a model generally regarded as the closest preclinical approximation to human RDEB skin. Xenograft experiments were conducted in two rounds, using 10 mice in each round. Eight mice in each round were treated with B-VEC, and two mice in each round were treated with topical saline control (total grafts, *n* = 16 B-VEC, *n* = 4 control). Control grafts (*n* = 4) had multiple areas of dermal–epidermal separation (Fig. 1k) not observed in xenografts following application of topical B-VEC (*n* = 16). These results are in agreement with a previous study that also reported dermal–epidermal separation in primary regenerated human RDEB xenografts and correction, following restoration of C7 expression due to treatment with an ex vivo cell-mediated *COL7A1* gene therapy^23^. Figure 1k also shows the immunofluorescence microscopy analysis of C7 expression in xenografts 5 days after topical B-VEC application. RDEB xenografts infected with control vector had no C7 expression. However, B-VEC-treated xenografts had robust linear detection of both the NC1 and NC2 domains of C7, indicating full-length C7 expression^24^.

**Extended Data Fig. 2:**
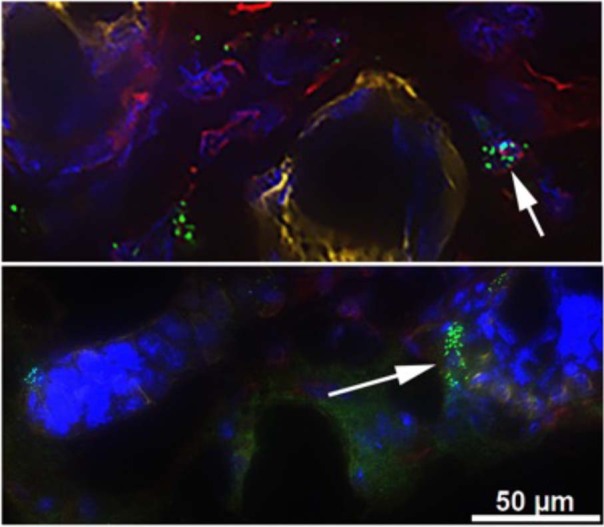
In vivo co-expression of B-VEC and keratinocyte, fibroblast-specific markers. Three days following B-VEC injection, RDEB mouse skin was biopsied and analyzed by dual label immunofluorescence microscopy using antibodies to the fibroblast marker vimentin (red, arrow upper panel), or antibodies to keratin 14 (yellow, arrow lower panel) combined with antibodies to ICP0. Scale bar is 50 μm. Representative of 3 mice; 3 treated back areas per mouse. B-VEC, beremagene geperpavec; RDEB, recessive dystrophic epidermolysis bullosa.

A crucial functional test of C7 is its assembly into basement membrane zone-associated AFs, a process requiring full-length C7 molecules containing both NC1 and NC2 domains. To determine full-length C7 expression following topical B-VEC treatment in human RDEB xenografts, biopsy tissue from the treated area was processed for immunoelectron microscopy using human-specific antibodies against the NC1 and NC2 domains. C7 NC1 and NC2 expression was detected as early as 3 days after B-VEC treatment and became more prominent 12 days after treatment. Correct ultrastructural localization of NC1 in the lamina densa (Fig. 1l) and NC2 approximately 300 nm below the lamina densa (Fig. 1l) was noted, consistent with previous structural localization studies of C7 NC1 and NC2 domains in AFs of normal human skin^25^. Taken together, these data demonstrate B-VEC-mediated *COL7A1* gene delivery, expression, and correct ultrastructural localization of functional, full-length C7 (inclusive of both NC1 and NC2) in RDEB mouse and primary human RDEB skin xenograft models, both of which closely recapitulate the RDEB skin phenotype. Furthermore, to demonstrate that C7 expression is attributable to B-VEC in vivo, the expression of the HSV-1 early protein, ICP0, was closely correlated with C7 expression in mouse skin after B-VEC injection (Extended Data Fig. 1). Following B-VEC treatment, in vivo co-localization of ICP0 expression with hair follicle keratinocytes, as well as dermal fibroblasts, was also noted (Extended Data Fig. 2).

### Molecular correction and treatment effect in patients with RDEB

#### Patients and treatment

**Fig. 2:**
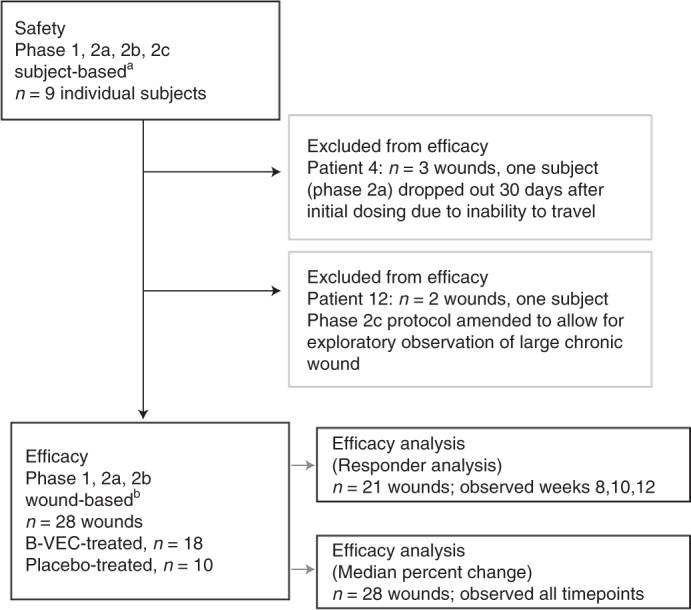
Trial profile. The safety population was evaluated considering an individual systemic patient response being the independent event (*n* = 9 patients). The efficacy assessments and analyses were conducted considering an individual wound response as the independent event (*n* = 28 wounds treated with B-VEC or placebo in the efficacy evaluable population: 18 B-VEC-treated wounds, 10 placebo-treated wounds). Responder analysis included patient wounds observed at weeks 8, 10 and 12 (21 wounds). ^a^Nine individuals of whom three participated in both 2a and 2b. One of nine individuals dropped out 30 days after initial dosing due to inability to travel. ^b^Intra-patient wound randomization: phase 1, 1:1; phase 2a, 2b, 2:1.

**Extended Data Fig. 3:**
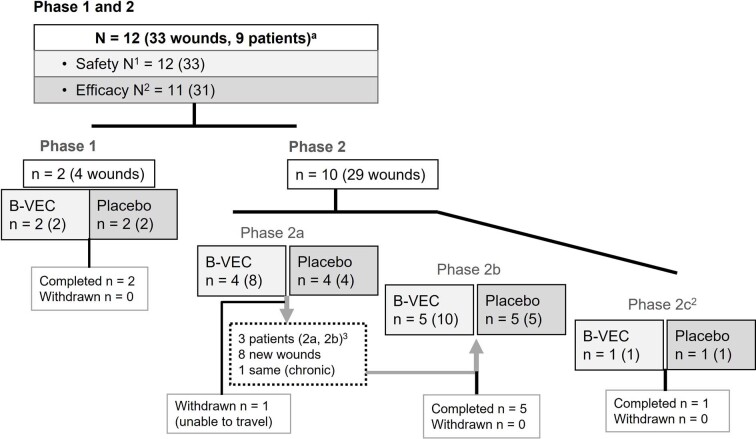
Overview of Phase 1 and 2 study design. ^a^Three (3) patients were enrolled into both Phase 2a and Phase 2b. These patients’ Phase 2b wounds were treatment-naïve except for chronic (5 year) dorsal foot wound on Pt 3. A wash-out period of 3 months passed between treatments in Phase 2a and 2b. Patients were enrolled in Phase 2a as Pts 3, 5, and 6, and in Phase 2b as Pts 11, 8, and 7, respectively. ^1^Phase 1, 2a, 2b, 2c; all interventions all wounds. ^2^Phase 1, 2a, 2b; randomized wounds (Pt 12, 2c considered separately for efficacy). ^3^Patients were enrolled in Phase 2a as Pts 3, 5, and 6 and in Phase 2b as Pts 11, 8, and 7, respectively. Pt, Patient.

Preclinical in vitro and in vivo data provided the scientific rationale for the initiation of a randomized, open-label, placebo-controlled phase 1 and 2 trial of topical B-VEC for treatment of RDEB conducted in an outpatient setting at Stanford University. The phase 1 and 2 study enrolled nine adult and pediatric patients (Fig. 2, Extended Data Fig. 3 and Extended Data Table 1) who had a clinical phenotype consistent with generalized RDEB^6^, confirmed *COL7A1* gene mutations, reduced C7 NC1, and absent C7 NC2 protein expression and absent AFs as assessed with immunofluorescence and immunoelectron microscopy. Baseline wound surface area ranged from 0.89 to 65.29 cm^2^ (Extended Data Table 2).

#### Safety

No deaths, serious or significant adverse events were reported (Extended Data Table 3). Of 129 topical B-VEC doses given in the trial, 21 adverse events were reported. One adverse event was moderate and was deemed unlikely to be related to the investigational product by the principal investigator. The remaining adverse events were mild: 13 adverse events were deemed unrelated to the investigational product, one was deemed unlikely to be related, four were reported as possibly related (fever, peculiar taste, rash, itching) and two were reported as probably related (application site discharge). All adverse events resolved during the study and none necessitated the reduction of B-VEC dosing or frequency. No vector shedding was reported (Supplementary Tables 1 and 2). Increased inflammation above and beyond what would be expected during normal wound healing was not seen in any of the B-VEC-treated wounds during any of the patient visits.

**Extended Data Fig. 4:**
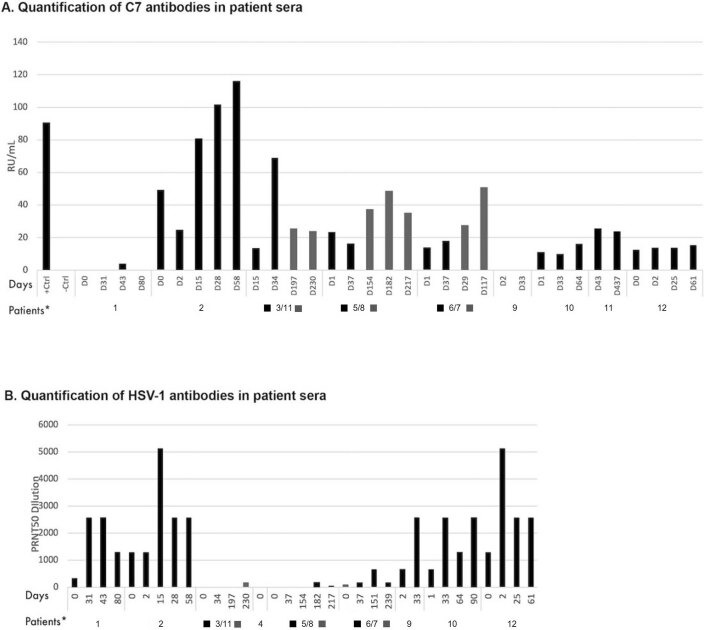
Patient antibody analysis. A. Quantification of collagen VII antibodies in patient sera. Patient sera was collected at baseline (day 0) or at indicated times following initiation of B-VEC treatment and analyzed for presence of collagen VII IgG antibodies using ELISA. A level greater than or equal to 20 reference units (RU) per mL is considered to be positive. B. Quantification of HSV-1 antibodies in patient sera. Patient sera was collected at baseline (day 0) or at indicated times following initiation of B-VEC treatment and analyzed for the presence of antibodies against HSV using a qualified Plaque Reduction Neutralization Test (PRNT), which utilizes B-VEC. *Patients 3, 5, and 6 subsequently re-enrolled as Patients 11, 8, and 7, respectively. B-VEC, beremagene geperpavec; ELISA, enzyme-linked immunosorbent assay; HSV-1, herpes simplex virus type 1; IgG, immunoglobulin G; RDEB, recessive dystrophic epidermolysis bullosa.

The HSV-1 serology status of the study patients was in line with the HSV-1 seropositivity rate of the US population (>50%)^26^. HSV-1 antibodies, when present, were detected at variable levels before and after B-VEC treatment (Extended Data Fig. 4) and did not affect the efficacy or safety. Some patients had baseline C7 antibodies (patients 2, 5) and some C7 ELISA titers increased after treatment (Extended Data Fig. 4); however, no tissue-bound skin immunoreactants were noted on immunofluorescence microscopy. The serum of patient 2 was subsequently found to be C7 antibody negative nearly 28 months later. Patient 12 had no C7 immunogenicity, despite having the greatest B-VEC exposure (8 × 10^8^ p.f.u. per dose every 1–2 days for two 25 day treatment cycles).

#### Findings following intervention

**Fig. 3:**
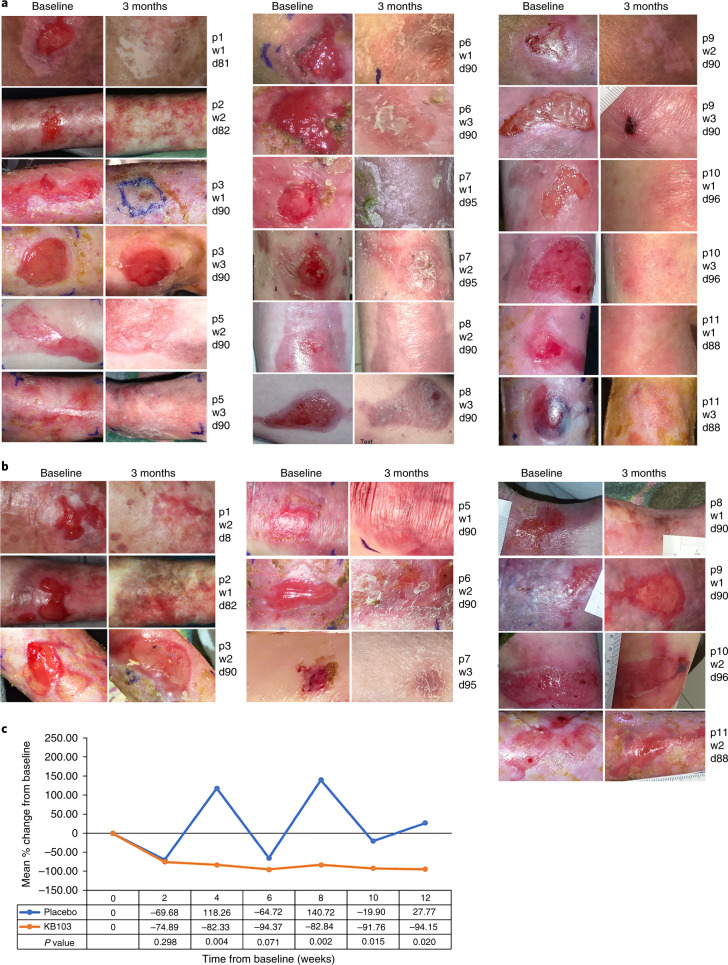
Assessment of RDEB patient skin wound healing following topical B-VEC or placebo. **a**,**b** Photos of B-VEC treated wounds (**a**) or placebo-treated wounds (**b**) on the indicated patients at baseline (before therapy) and at approximately 3 months after treatment. **c**, Mean percent change from baseline in the area of all target wounds, in patients 1–11 over the 12 week treatment period. KB103, B-VEC. *P* value was determined using the Wilcoxon rank-sum test. d, number of days from the start of therapy; p, patient number; w, wound number.

Images of all wounds studied in patients 1–11 are shown in Fig. 3, both at baseline and at 3 months after treatment with B-VEC or placebo. Closure in the B-VEC group was achieved in all wounds after 3 months, with the exception of a chronic (5 year) dorsal foot wound in patient 3. This wound had partial closure within 1 month of B-VEC treatment (Fig. 3; patient 3, wound 3), and complete closure upon re-treatment (Fig. 3; patient 11, wound 3). This wound remained healed throughout monitoring (8 months), highlighting the continued B-VEC effectiveness following repeated application. All other B-VEC-treated study wounds closed and remained healed for at least 3 months, while placebo wounds demonstrated a fluctuation of healing and re-blistering (Fig. 3). No healing rate differences were noted with respect to patient age or sex, B-VEC dosage or rates of application.

**Fig. 4:**
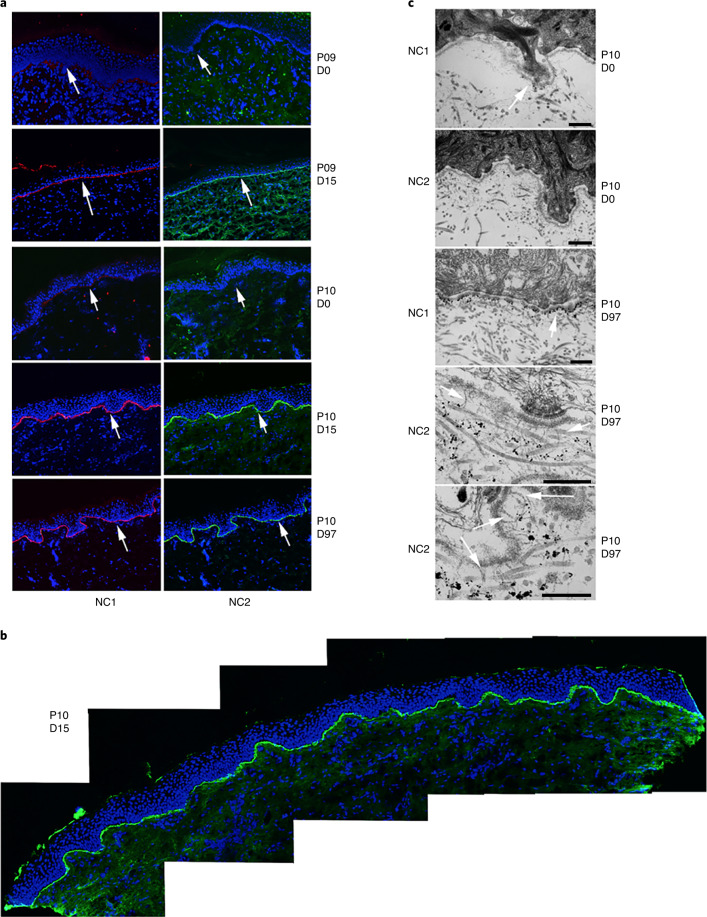
C7 expression and AF formation after B-VEC topical therapy. **a**, Indirect immunofluorescence analysis of C7 NC1 and NC2 expression in topical B-VEC-treated patient skin. In a subset of seven patients (Supplementary Tables 3 and 4), biopsies from intact B-VEC-treated skin were evaluated for C7 NC1 and NC2 expression. Representative images are shown from patients (P) 9 and 10 (collected on the indicated days (D)). These were analyzed using dual label immunofluorescence for expression of the C7 NC1 and NC2 domains using anti-NC1 antisera (red) and an anti-NC2 monoclonal antibody, LH24 (green), and counterstained with nuclear stain (blue). The arrows indicate the dermal–epidermal junction. **b**, Extended examination of C7 NC2 expression across an entire tissue section of topical B-VEC-treated patient skin. Multiple sections of a skin biopsy taken from a healed wound area 15 days after treatment with topical B-VEC were analyzed using immunofluorescence and tiled together to show the results across the entire tissue section. **c**, Immunoelectron microscopy^29^ of C7 NC1 and NC2 expression and AFs in B-VEC-treated patient skin. Representative images are shown from patient 10 (collected at the indicated times) and were analyzed with immunoelectron microscopy using antibodies to the C7 NC1 domain (NP185) and C7 NC2 domain (LH24). (C7 NC1 and NC2 expression was assessed in patients whose specimens were amenable to immunoelectron microscopy analysis (*n* = 3; Supplementary Table 4.) The arrow in the upper panel and center panel shows positive immuno-gold staining for the NC1 domain in the lamina densa region. Arrows in the lower two panels show the presence of mature banded AFs associated with immuno-gold staining for the NC2 domain approximately 300 nm from the lamina densa. Scale bars, 500 nm.

Wound closure analyses are given in Table 1 and Extended Data Table 4. Results and analysis reported are based upon observed data without imputed values for missing data. B-VEC-treated wounds were statistically significantly different from placebo wounds (*P* = 0.0026) based upon wound closure responder analysis; however, on both time to and duration of wound closure, the trend was numerically favorable towards B-VEC (Table 1). This observed treatment effect in B-VEC-treated wounds compared with the placebo control is suggestive of improved wound healing. Taken together with the evidence for molecular correction demonstrated in Fig. 4, this observed treatment effect suggests that, attributable to the expression of full-length C7, topical application of B-VEC improves RDEB wound healing, specifically, in the clinically meaningful terms of complete wound closure, time to wound closure and durability of wound closure.

**Table 1:** Summary of wound closure analyses

Complete wound closure^a^ at weeks 8, 10, or 12	Week	Wounds closed^a^ n (%)		% difference	
		B-VEC	Placebo^b^		
	8 (*n* = 21)	13/14 (93)	0/7 (0)	93	
	10 (N = 20)	11/14 (79)	2/6 (33)	46	
	12 (*n* = 19)	10/12 (83)	1/7 (14)	69	
Complete wound closure^a^ at weeks 8 and 10 or weeks 10 and 12	Weeks	Wounds closed^a^ n (%)		% Difference	
		B-VEC	Placebo		
	8 & 10 (N = 21)	11/14 (79)	0/7 (0)	79	
	10 and 12 (N = 19)	9/12 (75)	0/7 (0)	75	
Responder analysis: complete wound closure^a^ at weeks 8 and 10 or weeks 10 and 12	8 and 10 or 10 and 12 (*n* = 21)	11/14 (79)	0/7 (0)	% difference	*P* value^c^
				79	0.0026*
Time to complete closure^a,d^ (days)		Median (95% CI)^e,f^			
		B-VEC		Placebo	
		13.5 (8, 21)		22.5 (8, 64)	
Duration of closure^g^ (days)		103.0 (94, 118)		16.5 (0, 66)	

^a^Complete closure defined as ≥95% reduction in wound surface area from baseline.

^b^The placebo wound on patient 11 was not imaged, therefore the wound could not be confirmed as closed or open and patient 11’s wounds were not included in the responder analysis.

^c^*P* value based on McNemar’s test (one-sided).

^d^Time to wound closure was defined as the time from the first treatment to complete wound closure (≥95% reduction in wound surface area from baseline) observed for 2 consecutive weeks.

^e^The median estimate and the 95% confidence interval (CI) were derived using the Kaplan–Meier method.

^f^Numerically the trend favors B-VEC.

^g^Duration of wound closure was defined as the time from complete closure (≥95% reduction in wound surface area from baseline) to the first re-opening.

*Significant at *P* < 0.025.

The phase 1 protocol collected wound closure data at weeks 6 and 12 (no week 8 or week 10 datapoints), therefore the phase 1 patients (patients 1 and 2) were excluded from the responder analysis.

Patient 4 withdrew after initial dosing due to inability to travel.

Included in the analysis are data from week 8 for patients 3–10, week 10 for patients 3–10 (missing datapoint for patient 10, placebo), and week 12 for patients 3–10 (missing datapoints for patient 7, B-VEC). Results and analysis reported are based upon observed data without imputed values for missing data.

To further support these results, the mean percent change from baseline in wound surface area was analyzed (Fig. 3c). Comparisons used the Wilcoxon rank-sum test to demonstrate that the treatment effect of reduction in wound area is statistically significant (*P* < 0.025) at weeks 8, 10 and 12.

To validate wound closure assessments, 90 day wound images were evaluated by two blinded independent reviewers. Comparison of the unblinded principal investigator image assessments to this blinded review demonstrated 100% concordance.

Table 1 presents the analysis of time to and duration of wound closure using the Kaplan–Meier method. Time to wound closure was defined as the time from the first treatment to complete wound closure (defined as a ≥95% reduction in wound surface from baseline for 2 consecutive weeks). Duration of wound closure was defined as the time from complete closure to the first re-opening. Compared with placebo, B-VEC-treated wounds demonstrated a numerical trend of shorter time to wound closure and longer wound closure duration.

**Table 2:** Baseline characteristics of the study patients

Patient	Age (years)	Sex	Mutation 1	Mutation 2	NC1	NC2	AF	Collagen VII antibodies	Clinical diagnosis
1	36	Male	c.6527dupC	c.7485+5G>A	_+_	_	_	_	Generalized RDEB
2	28	Male	c.90delC	c.5048_5051 dupGAAA	_+_	_	_	_+_	Generalized RDEB
3	21	Male	c.1837C>T	c.5047C>T	_+_	_	_	_	Generalized RDEB
4	18	Male	c.1637-1G>A	c.5047CT	_+_	_	_	_	Generalized RDEB
5	13	Female	c.4478delA	c.5047C>T	_+_	_	_	_+_	Generalized RDEB
6	14	Male	p.G2233RfsX57	p.Q2488X	_+_	_	_	_	Generalized RDEB
7	15	Male	p.G2233RfsX57	p.Q2488X	_+_	_	_	_	Generalized RDEB
8	14	Female	c.4478delA	c.5047C>T	_+_	_	_	_+_	Generalized RDEB
9	21	Male	p.G2177AfsX29	p.G2177 AfsX29	_+_	_	_	_	Generalized RDEB
10	33	Female	c.6501G>A	c. 5048_5051 dupGAAA	_+_	_	_	_	Generalized RDEB
11	22	Male	c.1837C>T	c.5047C>T	_+_	_	_	_	Generalized RDEB
12	10	Male	c. 5009G>A	c.5132_5133ins5	_+_	_	_	_	Generalized RDEB

Patient 3 in phase 2a was enrolled in phase 2b as patient 11. Patient 5 in phase 2a was enrolled in phase 2b as patient 8. Patient 6 in phase 2a was enrolled in phase 2b as patient 7.

Characteristic of RDEB wounds, placebo-treated wounds fluctuated between natural open and healed states^27^, while B-VEC-treated wounds remained durably and consistently closed (Fig. 3). This wound closure durability is supportive of the effectiveness of B-VEC in promoting dermal–epidermal cohesion. Resistance to blister extension into a B-VEC-treated wound area is shown in Supplementary Videos 1 and 2, supportive of the effectiveness of B-VEC in promoting dermal–epidermal cohesion.

**Extended Data Fig. 5:**
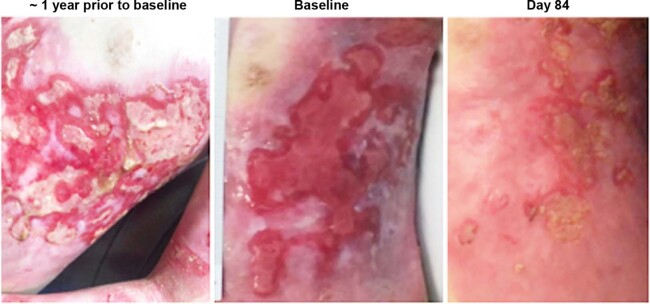
B-VEC treatment of large chronic wound. Results of Patient 12 treatment after two cycles of topical B-VEC therapy. Left panel shows presence of chronic wound 1 year prior to treatment. Center panel shows baseline appearance. Right panel shows the wound following B-VEC treatment. B-VEC, beremagene geperpavec.

**Extended Data Fig. 6:**
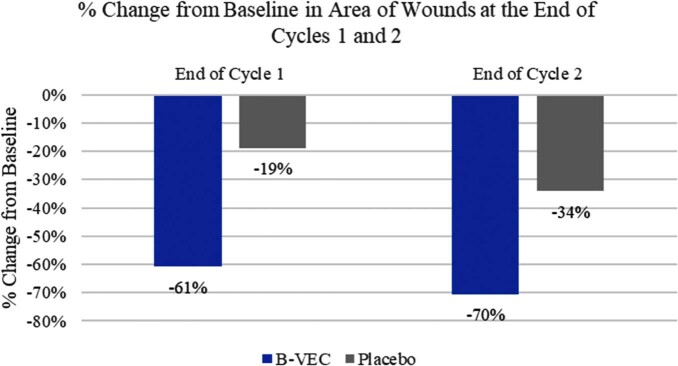
Patient 12, % change from baseline over time. The percent change in wound surface area for both B-VEC and placebo wounds was calculated over the course of treatment of Patient 12 and was plotted as a bar graph (end of cycle, % change area of wound from baseline). B-VEC, beremagene geperpavec.

Patient 12 was a 10-year-old boy who enrolled late in the study with large right and left lateral chronic (4 years) chest wounds that were much larger than those observed in the other patients. For that reason, his wound analysis was completed separately from the other patients (Extended Data Figs. 5, 6 and Extended Data Table 2). He completed two 25 day cycles of topical B-VEC (8 × 10^8^ p.f.u. per dose) every 1–2 days on his left chest (placebo on right chest) (Extended Data Table 2). Wound images 1 year prior to baseline, at baseline, and at 1 month after the last treatment are shown in Extended Data Fig. 5. By the end of the treatment, the B-VEC-treated wound was reduced by 70% from baseline whereas the placebo-treated wound had reduced by only 34% (Extended Data Fig. 6).

In a subset of seven patients (Supplementary Tables 3 and 4), biopsies from intact B-VEC-treated skin were evaluated for percent C7 NC1/NC2 expression compared with unaffected human skin (non-RDEB) using immunofluorescence and for AFs using immunoelectron microscopy, as previously described^22,28^. Some C7 NC1 and NC2 expression variability was noted in these post-treatment biopsies (Supplementary Table 3). However, most healed samples from this subset of seven patients had positive linear deposition indicative of full-length C7 expression as seen in representative images from patients 9 (day 15) and 10 (days 15 and 97) (Fig. 4a). NC2 expression in patient 10 on treatment day 15 was further analyzed by tiling multiple fields together to demonstrate continuous linear expression across the entire tissue section (Fig. 4b).

All patients who had biopsies prior to treatment had a lack of AFs at baseline (Table 2). Following B-VEC treatment, immunoelectron microscopy analysis^29^ showed C7 NC1 and NC2 expression and mature AFs (Fig. 4c and Supplementary Table 4) in patients whose specimens were amenable to immunoelectron microscopy analysis (*n* = 3). As shown in Fig. 4c, patient 10 lacked AFs and C7 NC2 staining, and had reduced C7 NC1 expression at baseline. Healed skin at treatment day 97 demonstrated increased C7 NC1 lamina densa localization and prominent C7 NC2 localization approximately 300 nm below the lamina densa (Fig. 4c), consistent with localization in normal skin^25^. These patient immunofluorescence and immunoelectron microscopy data suggest that B-VEC-mediated *COL7A1* gene delivery targets the proper cells and directs the expression of functional, full-length C7 (inclusive of both NC1 and NC2) at the correct location in the dermal–epidermal basement membrane. Providing even further evidence for expression of functional C7 and molecular correction, B-VEC-mediated *COL7A1* gene delivery promoted assembly of mature AFs.

## Discussion

Direct topical in vivo gene transfer with B-VEC fulfills a longstanding goal of direct in vivo gene therapy to the skin. The ability of B-VEC to be shipped off the shelf, applied topically in a local outpatient setting and repeatedly dosed on demand offers a number of advantages over earlier ex vivo approaches. These include eliminating the need for long-distance travel by fragile-skinned RDEB patients to specialized medical centers, making gene therapy accessible to patients who do not have access to specialized medical facilities, such as those living in underdeveloped countries. B-VEC therapy does not require patient biopsies for autologous cell engineering, nor does it require anesthesia or hospitalization. Instead, in vivo B-VEC topical gene therapy can be applied during routine dressing changes, minimizing any additional trauma and maximizing convenience to the patient.

Inflammation following in vivo treatment with gene therapy vectors has been a longstanding problem in the gene therapy field^30^. The most well-known example of this involved a patient fatality following the use of an adenoviral vector for the treatment of ornithine transcarbamylase deficiency^31^, in which the adenoviral vector activated innate immune responses and caused the acute release of inflammatory cytokines. The development of less immunogenic vectors such as adeno-associated virus and lentivirus has decreased but not eliminated inflammation, and repeated application of these vectors in vivo still usually requires concomitant systemic steroids. By contrast to previous in vivo studies, repeated topical application of B-VEC was not associated with any serious or significant adverse events. The natural immune-evasive properties of HSV-1, coupled with deletions in the immediate-early gene^20^, may explain the lack of inflammation associated with B-VEC and the lack of requirement for concomitant steroid or other anti-inflammatory medications, even after repeat applications. It may also explain why patient HSV-1 seropositivity has no discernible association with B-VEC efficacy. A previous ex vivo RDEB gene therapy study noted that circulating C7 antibodies did not interfere with C7 expression or wound healing and that the results of a C7-specific cytotoxic T cell assay were negative^10,11^. Similarly, in our clinical study patient 3’s wounds remained closed following B-VEC dosing, despite the presence of C7 antibodies, suggesting their lack of impact on B-VEC-mediated treatment efficacy.

Previous ex vivo cutaneous gene therapy trials limited retroviral exposure to cultured patient cells in vitro as a safety requirement^11,12^, given that random gene integration triggered cancers in some trials^32^. Although ex vivo retroviral gene transfer reduces systemic cancer risk, it does not eliminate oncogenic potential in transgene-expressing grafts, which is why these trials require long-term cancer surveillance^32^. Although adeno-associated virus is believed to involve less insertional mutagenesis risk^33^, the recent development of cancer in two patients on a lentivirus-mediated sickle cell disease trial has raised additional lentivirus cancer safety concerns^34^. HSV-1 vectors such as B-VEC, by contrast, are episomal and non-integrating, eliminating insertional oncogenesis risk.

B-VEC therapy may reduce cancer risk via mechanisms independent of insertional oncogenesis. Lethal squamous cell carcinoma, a devastating RDEB complication, arises from chronic wounding, inflammation and fibrosis^35^. Any C7 replacement therapy, such as topical B-VEC, if implemented early in the wounding process, has the potential to halt wound chronicity and fibrosis, which in turn could reduce lethal squamous cell carcinoma development, one of the most devastating complications in severe RDEB patients. Thus B-VEC therapy may not only improve but also prolong the lives of patients with RDEB. Further studies are needed to address this.

B-VEC treatment promoted molecular correction of RDEB skin concurrent with durable wound closure for 3 months or longer. In this study, wound healing durability and resistance to blistering was potentially aided by the long half-life of C7 in human skin^36^. Given that new trauma to other areas of skin can cause new blisters outside of previously treated wounds, it is anticipated that periodic B-VEC dosing of new wounds outside of previously treated wound areas will be necessary.

Whereas ectopic suprabasal C7 expression has been noted as a side effect in previous C7 gene replacement studies^6^, in the current study C7 was correctly localized solely to the dermal–epidermal junction. Many previous RDEB skin studies examined C7 NC1 expression but not the C7 NC2 domain. Because the NC2 domain of C7 is essential for AF assembly and function, NC1 expression by itself has not always been found to correlate with AF formation in previous clinical studies^8^. In contrast, this study examined both NC1 and NC2 expression as an indication of full-length functional C7 expression, both by immunofluorescence and immunoelectron microscopy. Although some variability was observed in NC1 and NC2 expression in this study, as has been noted in previous studies of *COL7A1* gene replacement^8,11^, when areas of C7 expression were noted, they were linear and continuous.

Keratinocytes and fibroblasts each contribute C7 to the dermal–epidermal basement membrane^37^, and a recent study has suggested that expression of C7 by both cell types may be required for optimal formation of normal AFs^38^. Although other *COL7A1* gene replacement approaches have targeted either keratinocytes^11^ or fibroblasts^13^, the ability of B-VEC to induce robust C7 expression in both cell types may offer a distinct advantage and may have contributed to the AF formation and wound healing seen in this study.

Limitations of this phase 1 and 2 trial include treatment of only open RDEB wounds with topical B-VEC as opposed to intact skin. B-VEC would not be expected to induce basal epidermal C7 expression when topically applied to intact skin because this vector has not been shown to efficiently penetrate intact skin in animal models. When possible, wounds of similar location and size were selected. However, due to the random nature of patient wounding and limited patient numbers, some wounds were not fully matched in location and size. Another limitation was the inability to correlate wound healing with patient-reported outcomes of severity and pain. The reasons for this we believe are twofold, in that patients on the study continued to use their regular pain medications, and the wounds treated in this early exploratory trial represented only a small subset of the total wounding and disability burden experienced by the patients. The secondary objective of investigator wound image assessment was redundant given that the primary objective of complete wound healing assessed by the investigator involved evaluation of both remote and on-site images taken at weeks 8, 10 and 12, and hence it was not re-analyzed.

In this exploratory phase 1 and 2 trial involving patients with RDEB, repeat topical B-VEC applications were associated with durable wound closure and full-length cutaneous C7 expression and AF assembly with minimal adverse events. Future therapeutic directions deserving to be investigated include topical delivery to mucosal surfaces affected in RDEB such as oropharynx, esophagus, or eyes, and HSV-1 transgene delivery for other genetic diseases. In total, the preliminary conclusions and treatment advances described here have far-reaching implications with the potential to broadly transform the gene therapy field. A phase 3 study of B-VEC is underway (ClinicalTrials.gov: NCT04491604).

## Methods

### Study design

This completed study was a single-center open-label, intra-patient, randomized, phase 1 and 2 placebo-controlled trial conducted at Stanford University (first patient enrolled on 6 May 2018, last patient enrolled on 3 September 2019). Eligible male and female patients were at least 6 years old and had been diagnosed with generalized RDEB using clinical guidelines. RDEB diagnosis was molecularly confirmed and C7 serology was determined for each patient, as previously described^11,39^.

Patients had at least two skin areas (0.89–65.29 cm^2^) with at least one wound in each area at the time of enrollment. Of nine patients screened, all were enrolled in this trial (Fig. 2). One patient withdrew at day 30 due to travel inability. Three patients enrolled into a later trial phase following a 3 month wash-out period and were considered independent for wound efficacy analyses. The principal investigator selected at least two and up to three target wounds meeting the inclusion criteria.

A complete randomized-block design, with each patient designated as a block to receive all conditions, was used to assign treatment. Investigator-assigned wound numbers were randomized using a pre-generated randomization schedule. Randomization schemes were generated via a customized program prior to the study, and randomization assignments for the treatment sites of each study patient were provided in sealed envelopes. Once the wound pairs were identified and labeled, the randomization envelope was opened and the assigned dose was then given.

One wound was treated with placebo and the other(s) treated with B-VEC (Extended Data Table 2). Following the safety board review of initial results, pediatric patients were included, and total dosing was increased to 2–8 × 10^8^ p.f.u. per wound per day.

The study was conducted in accordance with the 1996 Health Insurance Portability and Accountability Act and approved by the FDA Center for Biologics Evaluation and Research, and Stanford’s institutional review board (NCT03536143, registered 20 April 2018). Written informed consent was obtained from patients or from the patients’ legally authorized representatives for participation in the study, as well as for publication of images and videos of treated wounds obtained during the study. Patients received compensation to cover travel costs and meals for the day of the visit. A safety review committee conducted periodic assessments. Data were collected by the principal investigator and staff. Krystal Biotech prepared the statistical analysis plan and performed the statistical analysis. Version 9.1.3 of the SAS statistical software package was used to generate summaries, listings, graphs and statistical analyses. The principal investigator prepared the first manuscript draft. All authors interpreted the data, collaborated in preparing the manuscript, and vouch for data accuracy, completeness, and fidelity of the trial description to the protocol and the reporting of adverse events presented within the full text of this article. All authors and their institutions had confidentiality agreements with the sponsor.

### Study objectives

#### Phase 1

The primary objectives were to preliminarily assess the safety of topical B-VEC compared with placebo and to demonstrate evidence of molecular correction, that is, functional collagen VII (C7) expression and AFs, associated with B-VEC after treatment. The secondary objective was to preliminarily assess the effect of B-VEC on wound closure compared with placebo (wound surface area reduction, duration of wound closure). Other safety-related objectives were to assess changes in laboratory values and the condition of the patients (HSV and C7 antibodies, vital signs, physical examination).

#### Phase 2

The primary objective of the phase 2 trial was the further assessment of the effect of B-VEC on wound closure compared with placebo (wound surface area reduction, duration of wound closure, time to wound closure). An additional objective was to demonstrate evidence of molecular correction (functional C7 expression) associated with B-VEC after treatment.

Note that two additional secondary objectives were not reported in the manuscript. Patient-reported outcomes for pain and severity were not amenable to interpretation for two reasons: the patients on the study continued to use their regular pain medications, and the wounds treated in this early exploratory trial represented only a small subset of the total wounding and disability burden experienced by the patients. The investigator’s global assessment analysis was not reported due to redundancy relative to the primary objective. The investigator evaluated both remote and on-site images taken at weeks 8, 10 and 12 for wound healing, and these images were not re-analyzed.

### Inclusion criteria

To be eligible for inclusion, each patient fulfilled each of the following criteria: clinical diagnosis of the recessive form of dystrophic epidermolysis bullosa; age (initial three patients: 18 years or older; subsequent three patients: 5 years or older); willingness and ability to give consent or assent; confirmation of RDEB diagnosis on genetic testing, immunofluorescence and electron microscopy; LH24 antibody staining negativity and NC1 positivity (this criterion is applicable to the first two adults on the study; subsequent patients could be NC1 positive or negative); confirmed RDEB *COL7A1* mutations; at least one wound between 5 and 10 cm^2^ in wound area; and ability and willingness (in the opinion of the investigator) to understand the study, cooperate with the study procedures and to return to the clinic for all of the required follow-up visits.

### Exclusion criteria

Patients were excluded from the study if any of the following criteria were met: medical instability limiting ability to travel to the investigative center; the presence of medical illness expected to complicate participation and/or compromise the safety of this technique, such as active infection with HIV, hepatitis B or hepatitis C (as determined by hepatitis B surface antigen screening, detection of hepatitis C antibodies, or positive result of hepatitis C polymerase chain reaction (PCR) analysis); serum antibodies to type VII collagen demonstrated on ELISA, indirect immunofluorescence microscopy or Western blot, or cell-mediated immunity to ELISPOT (patients with negative results within 12 months of screening are eligible); active infection in the area that will undergo injection; evidence of systemic infection; known allergy to any of the constituents of the product; current evidence or a history of squamous cell carcinoma in the area that will undergo treatment; active drug or alcohol addiction; hypersensitivity to local anesthesia (lidocaine or prilocaine cream); receipt of a chemical or biological study product for the specific treatment of RDEB in the past 3 months; specific wounds that have previously been treated with investigational gene or cell therapy; the use of systemic antibiotics in the past 7 days; positive pregnancy test or current breast-feeding; clinically significant abnormalities (grade 2 or higher on the National Cancer Institute (NCI) toxicity scale) on laboratory tests before treatment (except for the following specific exclusionary laboratory threshold results, subject to approval or exemption by the epidermolysis bullosa physician: albumin < 1.7 g dl^−1^, leukocytes > 20,000 μl^−1^, hemoglobin < 7.5 g dl^−1^ (low hemoglobin is treated at the discretion of the investigators and the epidermolysis bullosa physician) and any additional exceptions made at the discretion of the investigators and the epidermolysis bullosa physician); and clinically significant abnormalities (grade 2 or higher on the NCI toxicity scale) identified through medical history and physical examination at day 0 (with the following exceptions: anorexia, can enroll up to grade 4 (inclusive); constipation, can enroll up to grade 2 (inclusive); dysphagia, can enroll up to grade 4 (inclusive); keratitis, can enroll up to grade 4 (inclusive); bone pain, can enroll up to grade 2 (inclusive); and any additional exceptions made at the discretion of the investigators and the epidermolysis bullosa physician).

### Clinical study treatment

The topical B-VEC gel or the placebo gel (produced according to Good Manufacturing Practice, GMP) was given dropwise by pipette uniformly across the surface area of the wound, without touching the wound itself, and not extending to normal skin surrounding the wound. Once B-VEC was evenly applied to the wound, a non-adhesive bandage (Tegaderm, 3M Health Care) was placed over the treated area, creating a uniform spread of a thin layer of gel across the wound surface, which was then covered by a secondary bandage for padding (Mepilex, Mölnlycke), and self-clinging gauze (Kerlix) was used to hold the dressings in place. The patients were advised to leave the dressings in place for 24–48 h. After bandage change, the bandage materials in direct contact with the treated wound were disposed of in a biohazard bag.

The vehicle was a heat sterilized 3% METHOCEL^TM^ aqueous gel, formulated in water, and manufactured to GMP standards by Velesco Pharmaceuticals. Several viscosities of this composition (ranging from 2% to 4%) were tested prior to the selection of this gel. In addition, other excipients including poloxamer 407 and F127 were evaluated at multiple concentrations. The 3% METHOCEL^TM^ formulation had optimal compatibility with B-VEC, based on the maximal stability of the investigational product (B-VEC + gel) at multiple temperatures (2–8 °C, ambient, 33 °C) and the in vivo safety and efficacy in animal models. A volume of ~0.2 ml with a 1:1 ratio of B-VEC and excipient gel was used for all B-VEC wounds ≥20 cm^2^. Placebo-treated wounds received the same volume of excipient diluted in 0.9% saline gel.

### Vector detection (blood and urine)

Blood and urine samples were collected before treatment and on all scheduled visits, as often as amenable (blood and urine sampling is uncomfortable for patients with epidermolysis bullosa) and assessed for the presence of B-VEC DNA using a validated quantitative PCR assay.

### Evaluation of immune response to HSV

Sera samples collected before treatment and during specific visits were evaluated for anti-drug antibodies against HSV with a qualified plaque reduction neutralization test (PRNT) that uses B-VEC. The PRNT assay determines the percent reduction in B-VEC-mediated plaque production in the presence of different dilutions of patient sera and is reported as PRNT_50_, which is the serum dilution at which a 50% reduction in plaques is observed. An increase in PRNT_50_ over time is suggestive of an increase in the presence of anti-HSV antibodies in the sera.

### Evaluation of immune response to collagen VII

Sera samples, collected before treatment and at intervals during the study, were evaluated with an anti-collagen VII ELISA (EA 1947-4801 G, Euroimmun), which determines the levels of human collagen VII immunoglobulin G (a level ≥20 relative units (RU) ml^−1^ is considered to be positive).

### Wound imaging

Images were taken using an iPhone camera system with the WoundMatrix application loaded on the iPhone device. WoundMatrix is a complete mobile wound management and telehealth solution for the secure capture, measurement and upload of images and data elements at the investigator–patient point of care. The WoundMatrix application requires a wound image with a predefined calibration marker (ruler in cm) placed on the same surface as the wound. The software allows the investigator or user to draw a line on the calibration marker that represents 1 cm. The software uses this line to count the number of pixels that the 1 cm represents. After the calibration process is complete, the software allows the user to trace the wound to determine its shape. Once the user completes the tracing, the software calculates the number of pixels (using predefined algorithms) for the irregular traced shape. The software uses this information to calculate the surface area using the number of pixels, the calibration line and the number of pixels in the irregular wound shape. All measurements are stored in the application and the investigator can create progress graphs to see the change in area, width and length over time. The application allows the investigator to create wound reports on specific patients to monitor the wound progression.

To obtain wound images, the investigator ensured that there was sufficient lighting and that the wound had been cleaned and dried. The calibration marker (ruler in cm) was placed next to the wound, avoiding direct contact and without causing any curvature to the ruler. The smart phone focused on the center of the wound and an image was captured holding the phone in landscape style straight on without the flash. Once the image was accepted, the investigator completed measurements through the WoundMatrix application downloaded to the smart phone. A calibration was completed by tracing 1 cm on the ruler. Width was determined by creating a line across the wound followed by determination of the length in the same fashion. The investigator traced the granulation, slough, necrotic tissues and other tissues (if applicable) and accepted the tracing. The image was saved and closed in the application, which provides a surface area calculation of the wound based on the area traced by the investigator using the 1 cm predefined reference calibration. If a wound was completely closed, no tracing is required, and the image would be saved for documentation. If the image is captured off-site, the patient or their caregiver captured an image using the WoundMatrix application on their personal device. The clinical site helped to download the software at the first visit and trained the patient or their caregiver in how to capture images. They were sent home with imaging guidance provided by WoundMatrix to assist them when they were off-site. The patient had limited access to and no control regarding the measurement of the images. When off-site the patient made sure that the lighting was similar to the on-site lighting and cleaned and dried the wounds before imaging. Once an image was captured using the predefined calibration marker (ruler in cm) the patient or their caregiver used the descriptive image given on the device, created by the investigator, noting which region to capture and select before imaging (that is, plantar (bottom) of the right foot). The image was uploaded to the investigator and they traced the wound through the application interface remotely to determine the surface area.

### Evaluation of wound areas

Post hoc statistical analysis was performed on the wound measurement data obtained from patients in the phase 1 and 2 clinical study. Data from randomized wounds were pooled for wound closure efficacy analysis of B-VEC compared with placebo. Wound closure data from patient 12 were not included in the analysis because the wound was a great deal larger than the others studied and the intent was to observe the impact of B-VEC on a large chronic wound.

### Main protocol changes

Three key protocol amendments corresponding to the study phases were made: phase 1 (v1.0, 19Apr2018) phase 2a (v2.2, 08Oct2018), phase 2b (v3.1, 12Mar2019) and phase 2c (v4.0, 01Aug2019) (Extended Data Table 1). The protocol versions represent intended design that was amended in consultation and with approval from oversight committees and regulatory authorities.

After two patients were enrolled into phase 1, the protocol was amended to increase the frequency and p.f.u. level of doses and to include patients aged 5 years and older (to v2.2). After four patients were enrolled into phase 2a, the protocol was amended to administer B-VEC every 2–3 days to correspond with bandage changes. The potential number of doses was increased and the dose level was set at 2 × 10^8^ p.f.u. per wound per treatment. Five patients were enrolled into phase 2b. Finally, the protocol was revised to version 4.0 to allow for the use of B-VEC for at least one large chronic wound. The potential dose level and the number of treatments were increased.

The phase 1 study enrolled two adults (Extended Data Table 1 and Extended Data Fig. 3). Two wounds ≤10 cm^2^ were selected per patient, and the wounds were randomized to receive either 1 × 10^8^ p.f.u. of B-VEC per dose or vehicle gel (placebo) on days 1 and 3, and on days 29 and 31. Patient 2 received additional topical B-VEC doses on days 15 and 45.

Following v1.0, the protocol was amended to increase the frequency and level of the doses from 1 to 3 × 10^8^ p.f.u. per wound per treatment, to increase the maximum wound size from 10 to 20 cm^2^, and to lower the minimum age of patients from 18 to 5 years.

The phase 2a study enrolled two adult and two pediatric patients (Extended Data Table 1 and Extended Data Fig. 3). Three wounds ≤20 cm^2^ were selected per patient: two wounds were randomized to receive 3 × 10^8^ B-VEC per dose and one was randomized to placebo. The protocol also allowed for dose escalation up to 6 × 10^8^ p.f.u. B-VEC per treatment day. Patients received topical B-VEC to wounds on days 1, 2, 3, 4, 5 and then at follow-up visits to the clinic. Following v2.2, the protocol was amended to administer B-VEC every 2–3 days to correspond with bandage changes and to increase the total number of doses. The dose level was set at 2 × 10^8^ p.f.u. per wound per treatment.

Phase 2b enrolled five patients (Extended Data Fig. 3 and Extended Data Table 1). Three patients who participated in phase 2a also participated in phase 2b (Extended Data Fig. 3 and Extended Data Tables 1, 2). New, untreated wounds were evaluated for the patients who rolled over into the phase 2b portion of the protocol except for one chronic wound in patient 3 who became patient 11. For the re-enrolled patients, a wash-out period of 3 months passed between treatments in phases 2a and 2b. These patients were enrolled in phase 2a as patients 3, 5 and 6 and in phase 2b as patients 11, 8 and 7, respectively (Extended Data Fig. 3). (Note that the patient identification numbers run from 1 to 12 because although nine patients enrolled, three enrolled in both 2a and 2b with unique identification numbers.) The primary differences between phase 2a and phase 2b were dose frequency and dose level. Phase 2b allowed for treatment at bandage changes, every 1–3 days, and the dose level was set at 2 × 10^8^ p.f.u. per wound per treatment (Extended Data Table 1). Following v3.2, the protocol was amended to administer B-VEC in two cycles. The age of inclusion was lowered to 2 years old. Wound areas were increased to up to 50 cm^2^, and with the increase in area the dose was increased to 6 × 10^8^ p.f.u. per wound per treatment (see protocol appendix for further details).

Phase 2c enrolled one pediatric male patient (Extended Data Fig. 3 and Extended Data Table 1). One large, chronic wound larger than 60 cm^2^ was treated with B-VEC. A similar wound, contralaterally located, was selected as the placebo wound. Due to the large surface area, 8 × 10^8^ p.f.u. B-VEC was topically administered to the wound per dose. The placebo wound was treated with vehicle control. The wounds underwent two cycles of topical treatment: cycle 1 was 25 days and consisted of 20 treatments, and cycle 2 was 24 days and consisted of 21 treatments.

In all phases of the study, topical B-VEC was applied during the wound dressing changes. Following dressing removal, wound surfaces were gently rinsed with sterile saline solution and patted dry with sterile gauze. B-VEC or placebo gel was applied over the surface of the wound (vehicle was a carboxymethylcellulose containing slightly viscous hydrogel) and the wound was overlaid with an occlusive dressing that was immobilized by wrapping with self-clinging gauze.

### Statistical analysis

Two wound types were assessed: a more common variant, recurrent wounds, which repeatedly close and re-blister; and a less common variant, chronic wounds, of 12 weeks or more duration^27,40^. In this study, patients could have either or both wound types. Considering the diverse wound nature and the local topical B-VEC treatment effect without systemic exposure, efficacy assessments and analyses were performed on wounds randomized to either B-VEC or placebo for each patient. Target wounds were imaged at trial visits and at home using the WoundMatrix phone application. Wound images were assessed by the unblinded principal investigator and by two independent blinded physician-dermatologists with bullous disease expertise.

Biopsies from intact B-VEC-treated skin were evaluated for percent C7 NC1 and NC2 expression compared with normal skin (non-RDEB) using immunofluorescence, and for AFs using immunoelectron microscopy, as previously described^11,28^.

Safety assessments were conducted with a single systemic patient response as the independent outcome. Adverse events and concomitant medications were assessed at each visit. Blood and urine were assessed for B-VEC DNA using a validated qPCR assay, and blood was assayed for C7 and HSV-1 antibodies (Extended Data Fig. 4 and Supplementary Tables 1, 2).

Because this was a first-in-human topical gene therapy exploratory study in an ultra-rare disease, a formal sample size calculation was not performed before commencement. However, preclinical work demonstrated the vector’s ability to direct sufficient C7 expression to reverse the disease. This enabled preliminary confirmation of the mechanism and efficacy in a small number of patients with confidence. The sample size was thus based on what was believed to be an adequate number of patients for this exploratory study to evaluate safety and efficacy.

After consultation with the regulatory agencies and upon further consideration, the original statistical analysis approach using the Cochran-Mantel-Haenszel test and the Breslow–Day test was modified considering the paired binary nature of endpoint measurements. To account for within-pair or within-patient correlations and prevent bias of treatment effect estimation and statistical inference, McNemar’s test was used for the primary analysis of efficacy data (responder analysis) reported in Table 1.

Given the 2 month cutaneous half-life of C7^36^ and the 4–8 week epidermis turnover rate^41^, the durability of B-VEC wound closure is anticipated to last around 12 weeks. This expectation informed the timepoint selection for analysis (landmark analysis). Based on a responder definition of complete wound closure (>95% reduction in wound surface area from baseline) for at least 2 consecutive weeks (weeks 8 and 10, or weeks 10 and 12), responder analysis comparison of the incidence of complete wound closure between B-VEC-treated wounds and placebo-treated wounds was performed on observed data (no imputed values). This responder analysis included only patients for whom datapoints for either the week 8 and 10 timepoint, and/or the week 10 and 12 timepoint were collected (patients 3 and 5–10). The phase 1 protocol did not include collection of wound measurements at weeks 8, 10 and 12 (patients 1 and 2). Patient 4 dropped out of the study after initial treatment and was unable to make the consecutive visits to the site hence no data could be collected. Patient 11’s B-VEC-treated wounds reduced in size (wound 1, responder, reduced 71% from baseline at week 8, 100% at weeks 10 and 12; wound 2, non-responder, reduced 56% from baseline at week 8, 75% at week 10, and 100% at week 12) but this patient’s data were not included in the responder analysis. Data were not collected for patient 11’s placebo-treated wound at weeks 8 and 10 due to an inability to remove the bandaging from the wound without causing significant discomfort to the patient. Patient 12’s wounds were outside of the size range of other wounds in the analysis and were excluded from the analysis.

In Fig. 3c, in which the mean percent change in wound area was assessed by treatment and timepoint, all observed wound closure data for patients 1–11 were analyzed and are presented with *P* values based on the Wilcoxon rank-sum test.

### Vector structure and description

B-VEC is a vector derived from wild-type HSV-1 through the deletion of two copies of the viral immediate-early gene ICP4, rendering the vector replication incompetent; in addition, ICP22 was also deleted to reduce the cytotoxic effect (the vector diagram is given in Supplementary Fig. 1). Two full-length copies of the human *COL7A1* gene, each with their own expression control elements, were then independently inserted into each ICP4 locus.

### Vector production

B-VEC was produced in a GMP-certified engineered cell bank infected with the B-VEC virus seed stock. Virus stocks were stored at −80 °C until just before use. The vehicle used was Dulbecco’s phosphate-buffered saline + 10% glycerol.

### Cell culture

Cells were previously isolated from skin biopsies taken as part of routine surgical or diagnostic procedures. All cells were cultured at 37 °C in 5% CO_2_. RDEB and normal human fibroblasts were grown in DMEM (Corning cellgro, Mediatech) supplemented with 10% FBS (PEAK Serum, cat. no. PS-FB1). RDEB and normal human keratinocytes were cultured in a 1:1 mix of defined keratinocyte serum-free medium (SFM; Life Technologies) and Medium 154 (Cascade Biologics) at 37 °C in a humidified 5% CO_2_ incubator.

### Virus infection

Viral aliquots were stored at −80 °C and thawed 10 min before infection. Multiplicity of infection was calculated from the virus titer and target cell number and the appropriate volume of virus stock was diluted in DMEM and incubated with the target cells for 2 h at 37 °C. Virus was then removed, inactivated with acetic acid and discarded, and fresh media was supplied to target cells after washing twice with pre-warmed media.

### Western blot analysis

The cell lysate was prepared as follows: 8 × 10^5^ fibroblasts or keratinocytes were plated in a 100 mm dish to achieve 70–80% confluence the following day. At 48 h after infection, cells were lysed with radioimmunoprecipitation assay buffer^42^. Lysate was centrifuged at 13,500 ×*g* for 5 min at 4 °C, and the supernatant was mixed with a 6x Laemmli loading buffer. Before loading onto the SDS–PAGE gel, the samples were heated for 5 min at 95 °C. For C7 detection, 5–30 μg protein was loaded onto a 6% acrylamide gel. The primary antibodies used were C7 polyclonal rabbit antibody (Sigma prestige Ab, cat. no. HPA042420), GAPDH and β-actin (Santa Cruz Biotechnology). Resolved proteins were transferred onto nitrocellulose membrane with a BioRad Trans-Blot-Turbo (BioRad), blocked in PBS and 0.1% Tween with 5% milk or 5% BSA according to the requirements of the primary antibody, and incubated overnight with the primary antibody. After incubation with IgG-HRP-conjugated secondary antibody (Santa Cruz Biotechnology), the membrane was incubated with Pierce ECL western blotting substrate (ThermoFisher Scientific) and exposed to CL-XPosure X-ray film (ThermoFisher Scientific). C7 was quantified by densitometry (ImageJ v1.52), using a known concentration of purified recombinant C7 (donated by Krystal Biotech) for comparison.

### Protein quantification

Total cell lysates were quantified with the Pierce BCA (bicinchoninic acid) Protein Assay kit (ThermoFisher Scientific) and proteins were loaded onto an SDS–PAGE gel. The Western blot signal was quantified with ImageJ v1.52. Type VII collagen was quantified relative to the non-treated control, to GAPDH, and to purified recombinant type VII collagen supplied by Krystal Biotech.

### qRT–PCR

RNA was isolated using the RNeasy Mini Kit (Qiagen) according to the manufacturer’s instructions. RNA extractions were quantified using a NanoDrop Spectrophotometer (ThermoFisher Scientific), and 1.5 μg RNA was used for complementary DNA synthesis with the SuperScript III First-Strand Synthesis System (ThermoFisher Scientific). For detection of *COL7A1* expression the following primers were used: forward primer, GGCTGCAATTCTCCATGTGG; reverse primer, CTGTGAGGCAACTCGCTTCA. For ACTB amplification the following primers were used: forward primer, CATGTACGTTGCTATCCAGGC; reverse primer, CTCCTTAATGTCACGCACGAT. For qPCR, the SYBR Select Master mix (Life Technologies) was used and cDNA samples were diluted 1:25 to serve as the template. Experiments were performed in triplicate.

### Animals

A colony of homozygous *Col7a1*^flNeo^ mice, which are a strain expressing only 10% of the amount of murine type VII collagen found in normal mouse skin, was used. These mice have a phenotype similar to severe generalized human RDEB^22^. These were established from a breeding pair donated by L. Bruckner-Tuderman (Freiberg, Germany). Mice were genotyped with DNA extracted from an ear punch tissue sample. PCR analysis detected the presence of a loxP site upstream of exon 2 of *Col7a1*. Wild-type mice show a band at 269 bp and hypomorph mice show the band at 435 bp, while heterozygous mice have both. Balb/C mice were obtained from The Jackson Laboratory. For xenografting, NOD/SCID mice were used (NOD.CB17-PrkdcSCID/J mice; stock 001303; The Jackson Laboratory). All procedures used in the protocol were in compliance with applicable animal welfare acts and were approved by the Stanford Institutional Animal Care and Use Committee (IACUC). Animals were housed under the following conditions: a 14 h–10 h light–dark cycle, temperature of 18–23 °C and 40–60% humidity.

### Preclinical B-VEC viral vector application

Before and during the test article treatment, mice were maintained under inhalation anesthesia using 2% isoflurane. Eye ointment (Puralube Vet) was applied on the eyes to prevent dryness. The back and flank area was shaved using an electrical pet clipper and the area was wiped with an alcohol wipe. The viral vector was kept on dry ice until 10–15 min before injection, thawed at room temperature, and used within 15–30 min of thawing.

For intradermal injections the animal’s back was shaved and the mouse was injected at four sites on the back, consisting of one control vehicle injection and three B-VEC injections. Intradermal injections were performed using the Mantoux technique with a 31 G BD SafetyGlide 0.3 ml insulin syringe, ensuring creation of a superficial wheal at each site. Up to four intradermal injections were given to the back of each mouse at the doses specified in the Results section. The edges of the wheal were marked with a permanent marker. All mice received a dose of 4.6 × 10^7^ p.f.u. per 50 μl per injection site by intradermal injection. In some experiments the mice received a second injection at the same four sites on day 3. For RDEB mouse topical applications, a full-thickness 8-mm-diameter wound on the animal’s mid-back was locally anesthetized and covered with Tegaderm (3M Health Care), and the virus was injected into the space between the Tegaderm and wound surface. The Tegaderm was preserved on a mouse until tissue collection (2, 7, 10, 14 days). For xenograft topical applications the virus was injected into the Telfa non-adherent pad directly in contact with the xenograft surface.

### 3D xenograft preparation

Primary fibroblasts and keratinocytes cultured from a skin biopsy of patient with RDEB lacking C7 NC1 and NC2 expression were used to seed on porcine devitalized dermis. To produce the composite skin graft preparation, porcine devitalized dermis was cut into 2 × 2 cm square pieces and left to dry under aseptic conditions at the bottom of the 6-well tissue culture plate for 2 h. Fibroblasts were seeded from the bottom of the dermis and grown for 3 days. At day 4, 5 × 10^6^ keratinocytes were resuspended in a maximal volume of 150 ml of 50/50 V and placed in the middle of the dermis to avoid spillage of the cells onto the plate surface. The dermis squares were placed on special frames, to allow nutrients access to both sides of the graft. After another 2 h, wells were filled with 2.5 ml keratinocyte growth medium and allowed to grow further for at least 5 days. Next, the composite skin graft was removed from the plate with forceps touching only one side of the graft and used immediately for transplantation as described in the following section.

### Graft transplantation

Mouse grafting was performed as previously described^43^. In brief, 6–8-week-old mice were anesthetized with inhalation of 1.5–2% isofluorane. After shaving the hair from the mouse back, a rectangular region of mouse skin (~1.6 × 1.4 cm) was removed with scissors and the composite graft was sutured to the mouse skin using an interrupted stitch technique. Both dermal and epidermal components were kept moist with sterile culture medium throughout the transplantation procedure. Non-adherent dressing (TELFA; Tyco Healthcare/Kendall) was cut into 2 × 2 cm squares and placed on top of the graft. Next, Tegaderm dressing (3M Health Care) was wrapped around the mouse and then covered with a Coverlet adhesive dressing (BSN Medical). Last, a double layer of CoFlex (Andover Healthcare) was wrapped around the mouse. The mouse was subcutaneously injected with carprophen analgesic in the vicinity of the graft. The dressing was removed 10–14 days after grafting and the grafts were then further characterized.

### Tissue collection

Before tissue collection, animals were euthanized by CO_2_ inhalation followed by cervical dislocation. The back area was shaved, as needed to identify the marked injection sites or topical application areas, and the treated sites were biopsied using sharp scissors. Approximately one-half was snap-frozen in liquid nitrogen for qPCR analysis and the other half was cryopreserved in optimal cutting temperature (OCT) compound.

### Histologic analysis

The cryopreserved tissues in OCT compound were sectioned at a thickness of 5–8 µm and left to air dry for up to 1 h. The slides were dipped in 100% MeOH for 10 min at −20 °C and left to air dry. Methanol-fixed sections were rehydrated in PBS for 5 min at room temperature. The slides were analyzed using hematoxylin and eosin (H&E) staining or indirect immunofluorescence microscopy. H&E staining was performed using standard procedures with Weigert’s modified hematoxylin (HEXWHALE 100) and Eosin Y solution (HT110116). Indirect immunofluorescence microscopy was performed as previously described^44^. Primary antibodies were incubated for 16 h at 4 °C after 1 h of incubation at 20 °C with fluorescence (4′,6-diamidino-2-phenylindole) for nuclei staining. The stained sections were mounted with mounting media (Fluoromount G, cat. no. 0100-01, Southern Biotech) and overlaid with a glass coverslip. Antibodies used for the staining are listed in Supplementary Table 5. All imaging and image processing (fluorescent and H&E), including tiling of the images, was done using AxioVision SE64 v4.9.1 by Zeiss.

### Reporting Summary

Further information on research design is available in the Nature Research Reporting Summary linked to this article.

## Online content

Any methods, additional references, Nature Research reporting summaries, source data, extended data, supplementary information, acknowledgements, peer review information; details of author contributions and competing interests; and statements of data and code availability are available at 10.1038/s41591-022-01737-y.

### Supplementary information

Supplementary Information Supplementary Tables 1–5, Supplementary Fig. 1

Reporting Summary

Supplementary Video 1 Functional demonstration of dermal–epidermal cohesion following B-VEC therapy

Supplementary Video 2 Functional demonstration of dermal–epidermal cohesion following B-VEC therapy

### Source data

Source Data for Figs. 1B, 1C, 1G, 1H Statistical source data

Source Data for Fig. 1d Unprocessed Western blots

## Figures and Tables

**Extended Data Table 1:**
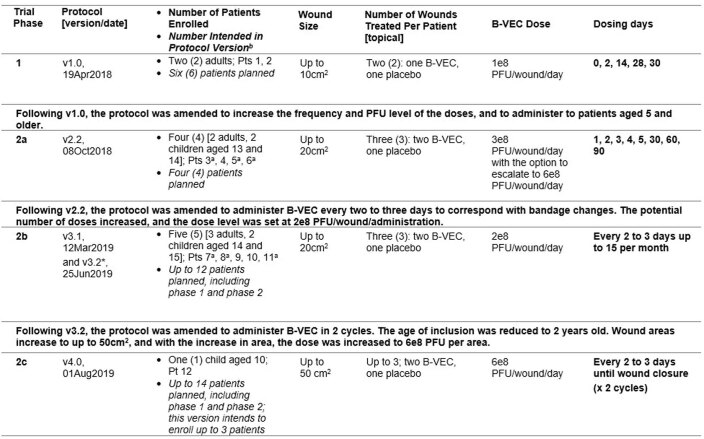
Summary of protocol phases/versions and amendments submitted to FDA and oversight committees

B-VEC has been administered to nine (9) patients, in twelve (12)^a^ siloed participant treatment applications. Patients (IDs: Pts 1-12)^a^ enrolled separately onto one of 4 versions of the Phase 1 and 2 protocol. A summary of the protocols and patients enrolled are presented below. ^a^Three patients were enrolled into both Phase 2a and Phase 2b. These patients’ Phase 2b wounds were treatment-naïve except for a chronic (5 year) dorsal foot wound on Pt 3. A wash-out period of 3 months passed between treatments in Phase 2a and 2b. Patients were enrolled in Phase 2a as Pts 3, 5, and 6 and in Phase 2b as Pts 11, 8, and 7, respectively. Pts # refer to patient identification numbers 1-12, nine total patients enrolled, three enrolled in both 2a and 2b with unique identification number. ^b^Protocol versions represent intended design which was amended in consultation and with approval from oversight committees and regulatory authorities; protocol amendments correspond to Phases (1, 2a, 2b, 2c). *v3.2 clarified the long-term follow-up. B-VEC, beremagene geperpavec; Pt, Patient.

**Extended Data Table 2:**
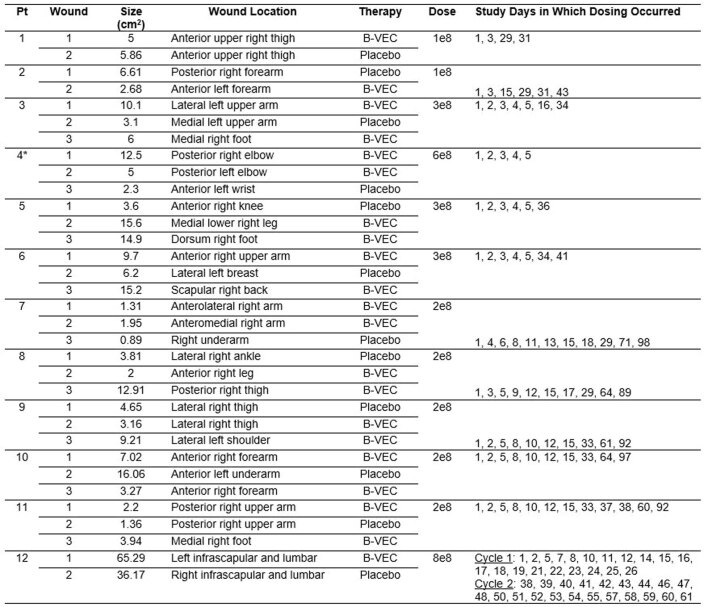
Patient wound disposition and treatment

Note: Patient 3 in Phase 2a was enrolled in Phase 2b as Patient 11. Patient 5 in Phase 2a was enrolled in Phase 2b as Patient 8. Patient 6 in Phase 2a was enrolled in Phase 2b as Patient 7.

*Patient 4 enrolled in Phase 2a and dropped out after initial dosing.

**Extended Data Table 3:**
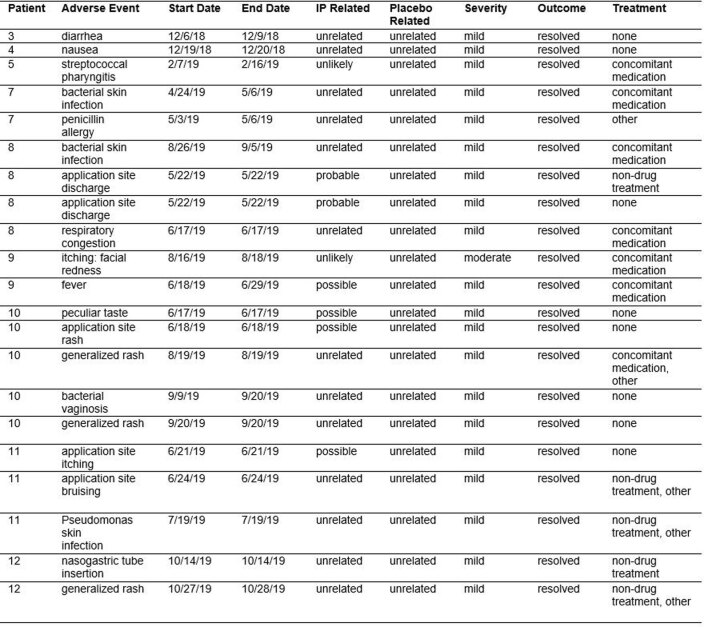
Adverse events list

**Extended Data Table 4:**
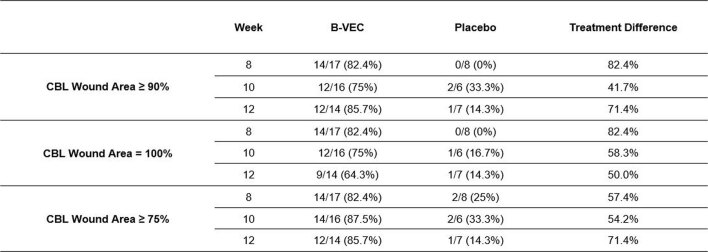
Supplemental analysis of reduction in wound surface area

CBL, change from baseline.

## Data Availability

All requests for data will be reviewed by the leading clinical site, Program in Epithelial Biology and Department of Dermatology, Stanford University School of Medicine, and the study sponsor, Krystal Biotech, to verify whether the request is subject to any intellectual property or confidentiality obligations. Requests for access to the patient-level data from this study can be submitted via email to medinfo@krystalbio.com with detailed proposals for approval. A signed data access agreement with the sponsor is required before accessing shared data. Source data are provided with this paper. The availability of the B-VEC vector is subject to a material transfer agreement.

## Extended data

are available for this paper at 10.1038/s41591-022-01737-y.
