# Supplementary material for: In vivo topical gene therapy for recessive dystrophic epidermolysis bullosa: a phase 1 and 2 trial
Source: Nat Med. 2022 Mar 28;28(4):780–8. doi: 10.1038/s41591-022-01737-y (PMC9018416; doi:10.1038/s41591-022-01737-y)
Supplement: Supplementary file 1 — Supplementary Tables 1–5, Supplementary Fig. 1 [file 41591_2022_1737_MOESM1_ESM.pdf]

nature portfolio

https://doi.org/10.1038/s41591-022-01737-y

Supplementary information

In vivo topical gene therapy for recessive dystrophic epidermolysis bullosa: a phase 1 and 2 trial

In the format provided by the authors and unedited

Supplementary Information

In vivo topical gene therapy for recessive dystrophic epidermolysis bullosa: a phase 1 and 2 trial

I Gurevich,[1] P Agarwal,[2] P Zhang,[2] JA Dolorito,[1] S Oliver,[2] H Liu,[2] N Reitze,[2] N Sarma,[2] IS Bagci,[1] K Sridhar,[1] V Kakarla,[1] VK Yenamandra,[1] M O'Malley,[2] M Prisco,[3] SF Tufa,[4] DR. Keene,[4] AP South,[3] SM Krishnan,[2] and MP Marinkovich.[1,5]*

[1] Program in Epithelial Biology and Department of Dermatology, Stanford University School of Medicine, Stanford, CA

[2] Krystal Biotech, Pittsburgh, PA

[3] Sidney Kimmel Medical College, Thomas Jefferson University, Philadelphia, PA

[4] Microscopy Unit, Shriners Hospital for Children, Portland, OR

[5] Veterans Affairs Medical Center, Palo Alto, Stanford, CA

*Corresponding author MP Marinkovich, 455 Broadway St., Discovery Hall, Room D126, Redwood City CA 94063; office 650-498-5425; fax 650-721-7152: email: mpm@stanford.edu

This PDF file includes:

Table S1. Phase 1 vector shedding analysis (blood and urine)

Table S2. Phase 2b vector shedding analysis (blood and urine)

Table S3. Immunofluorescence microscopy of patient biopsies

Table S4. Immuno-electron microscopy of patient biopsies

Table S5. Antibodies used for immunofluorescence microscopy

Fig. S1. B-VEC vector design

Video S1A, S1B. Functional demonstration of dermal-epidermal cohesion following B-VEC therapy

Table S1. Phase 1 vector shedding analysis (blood and urine)

Patient	Sample Type	Study Day	Assay Call
1	Blood	0	<LOD
	Blood	2	<LOD
	Blood	28	Negative
	Blood	30	Negative
	Blood	42	<LOD
	Urine	0	<LOD
	Urine	2	Negative
	Urine	14	<LOD
2	Blood	0	<LOD
	Blood	2	<LOD
	Blood	14	<LOD
	Blood	28	Negative
	Blood	30	Negative
	Urine	0	Negative
	Urine	2	Negative
	Urine	14	Negative
	Urine	28	Negative
	Urine	30	<LOD

Table S2. Phase 2b vector shedding analysis (blood and urine)

Patient	Sample Type	Study Day	Assay Call
7	Blood	117	< LOD
8	Blood	60	Negative
9	Blood	30	Negative
10	Blood	30	< LOD
11	Blood	30	< LOD
7	Urine	117	< LOD
8	Urine	60	< LOD
10	Urine	30	< LOD
	Urine	60	< LOD
11	Urine	30	Negative
	Urine	60	< LOD

Table S3. Immunofluorescence microscopy of patient biopsies*

Patient	NC1 IF	NC2 IF
1 – baseline	10	0
1 – week 4	80	80
2 – baseline	20	0
2 – week 8	80	80
3 – baseline	10	0
3 – week 4	80	70
4[a] – baseline	10	0
5 – baseline	5	0
5 – week 4	30	0
6 – baseline	10	0
6 – week 4	90	90
7 – baseline	10	0
7 – week 8	90	90
8 – baseline	5	0
8 – week 1	30	30
8 – week 4	20	0
9 – baseline	5	0
9 – week 2	80	90
9 – week 4	80	90
10 – baseline	10	0
10 – week 2	100	100
10 – week 13	100	100
11[b] – baseline	10	0
12[b] – baseline	10	0

Numbers are expressed as percent fluorescence intensity compared to normal human (non-RDEB) skin.

[a]Patient 4 withdrew due to inability to travel and was unavailable for post treatment skin biopsy.

[b]Patients 11 and 12 declined post treatment skin biopsies.

Table S4. Immuno-electron microscopy of patient biopsies*

Patient ID	NC1 IEM	NC2 IEM
2 – baseline	-	-
2 – week 2	+	-
2 – week 8	++	+
9 – baseline	-	-
9 – week 2	++	+
10 – baseline	-	-
10 – week 2	++	++
10 – week 13	++	++

++ 75-100% of normal skin
+ 25-75% of normal skin
- less than 25% of normal skin

*Post-treatment biopsies for patients 1, 3, 5 (8), and 6 (7) could not be analyzed due to dermal-epidermal separation occurring during overnight transport of unfixed IEM skin biopsies.

*Patients 11 and 12 declined skin post treatment skin biopsies.

*Patient 4 dropped out and was unavailable for post treatment skin biopsy.

Table S5. Antibodies used for immunofluorescence microscopy

Target molecule	Origin/isotype/type	Source	CAT#
human type VII collagen	Rabbit, monoclonal, IgG	Sigma	HPA042420
integrin alpha 6 (clone goH3)	Rat, IgG	BD Biosciences	555734
human type VII collagen NC1 (clone NP185)	Mouse monoclonal IgG	Produced in Lab	Reference[44]
human type VII collagen NC2 (clone LH24)	Mouse monoclonal IgM	Produced in Lab	Reference[11]
Anti-laminin 332 antisera (pKal)	Rabbit polyclonal IgG	Produced in Lab	Reference[42]
Anti-HSV-1 ICPO	FITC conjugated Mouse monoclonal IgG	Santa Cruz	sc53070
Anti-Keratin 14	Mouse monoclonal IgG	Novus	nbp2-47720af647
Anti-Vimentin<br>For Xenografts<br>For Mouse skin	Rabbit monoclonal IgG<br>Chicken polyclonal IgK	ABCAM<br>ABCAM	ab16700<br>ab24525

Fig. S1. B-VEC vector design

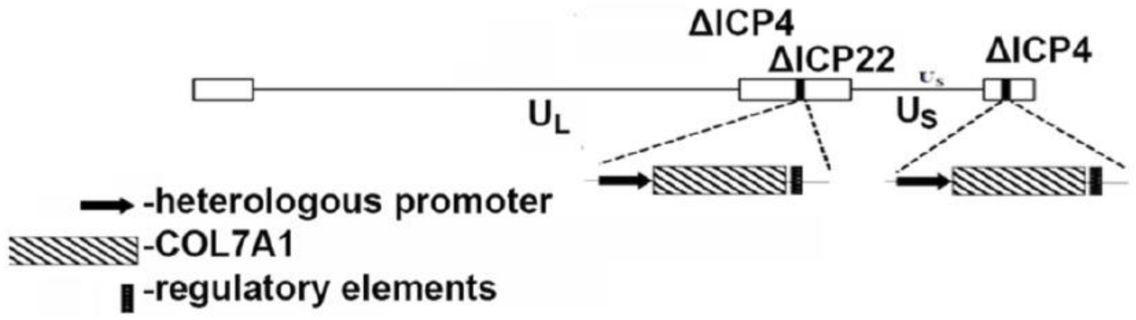

Bercolagene telserpavec, B-VEC, is a vector derived from wild type HSV-1 through the deletion of two copies of the viral immediate early gene ICP4, rendering the vector replication incompetent; in addition, ICP22 was also deleted to reduce cytotoxic effect. Two full-length copies of the human *COL7A1* gene, each with their own expression control elements, were then independently inserted into each ICP4 locus.

Video S1A, S1B. Functional demonstration of dermal-epidermal cohesion following B-VEC therapy.

These videos show the healed area of wound 1 on the right forearm of patient 10 six months following B-VEC therapy. The patient had suffered trauma to the area which created a blister, however the area treated by B-VEC has remained intact, despite all the skin around the healed wound blistering around the periphery of the treated area.
