# Supplementary material for: In vivo topical gene therapy for recessive dystrophic epidermolysis bullosa: a phase 1 and 2 trial
Source: Nat Med. 2022 Mar 28;28(4):780–8. doi: 10.1038/s41591-022-01737-y (PMC9018416; doi:10.1038/s41591-022-01737-y)
Supplement: Supplementary file 2 — Reporting Summary [file 41591_2022_1737_MOESM2_ESM.pdf]

nature portfolio

Corresponding author(s): M. Peter Marinkovich, M.D.

Last updated by author(s): January 18, 2022



# Reporting Summary

Nature Portfolio wishes to improve the reproducibility of the work that we publish. This form provides structure for consistency and transparency in reporting. For further information on Nature Portfolio policies, see our Editorial Policies and the Editorial Policy Checklist.

## Statistics

For all statistical analyses, confirm that the following items are present in the figure legend, table legend, main text, or Methods section.

| n/a | Confirmed | |
|---|---|---|
| ☐ | ☒ | The exact sample size (*n*) for each experimental group/condition, given as a discrete number and unit of measurement |
| ☐ | ☒ | A statement on whether measurements were taken from distinct samples or whether the same sample was measured repeatedly |
| ☐ | ☒ | The statistical test(s) used AND whether they are one- or two-sided<br>*Only common tests should be described solely by name; describe more complex techniques in the Methods section.* |
| ☐ | ☒ | A description of all covariates tested |
| ☐ | ☒ | A description of any assumptions or corrections, such as tests of normality and adjustment for multiple comparisons |
| ☐ | ☒ | A full description of the statistical parameters including central tendency (e.g. means) or other basic estimates (e.g. regression coefficient) AND variation (e.g. standard deviation) or associated estimates of uncertainty (e.g. confidence intervals) |
| ☐ | ☒ | For null hypothesis testing, the test statistic (e.g. *F*, *t*, *r*) with confidence intervals, effect sizes, degrees of freedom and *P* value noted<br>*Give P values as exact values whenever suitable.* |
| ☒ | ☐ | For Bayesian analysis, information on the choice of priors and Markov chain Monte Carlo settings |
| ☒ | ☐ | For hierarchical and complex designs, identification of the appropriate level for tests and full reporting of outcomes |
| ☒ | ☐ | Estimates of effect sizes (e.g. Cohen's *d*, Pearson's *r*), indicating how they were calculated |

*Our web collection on statistics for biologists contains articles on many of the points above.*

## Software and code

Policy information about availability of computer code

| | |
|---|---|
| Data collection | Data was collected using case report forms and data was entered into an electronic data capture system. All imaging and image processing (fluorescent and H&E), including tile images was done using AxioVision SE64 Rel. 4.9.1 Software by Zeiss. Densitometry of Western Blots was performed using ImageJ software v1.52. Images were taken using an iPhone camera system with the Wound Matrix application that was loaded on the iPhone device. |
| Data analysis | Version 9.1.3 of SAS statistical software package was used to generate summaries, listings, graphs and statistical analyses. Figure 1 data plots, including error bars and p values, were generated using GraphPad Prism software v8.3.0. |

For manuscripts utilizing custom algorithms or software that are central to the research but not yet described in published literature, software must be made available to editors and reviewers. We strongly encourage code deposition in a community repository (e.g. GitHub). See the Nature Portfolio guidelines for submitting code & software for further information.

## Data

Policy information about availability of data

All manuscripts must include a data availability statement. This statement should provide the following information, where applicable:

- Accession codes, unique identifiers, or web links for publicly available datasets
- A description of any restrictions on data availability
- For clinical datasets or third party data, please ensure that the statement adheres to our policy

All requests for data will be reviewed by the leading clinical site, Program in Epithelial Biology and Department of Dermatology, Stanford University School of Medicine, and the study sponsor, Krystal Biotech, to verify whether the request is subject to any intellectual property or confidentiality obligations. Requests for

access to the patient-level data from this study can be submitted via email to medinfo@krystalbio.com with detailed proposals for approval. A signed data access agreement with the sponsor is required before accessing shared data. Source data are provided with this paper.

# Field-specific reporting

Please select the one below that is the best fit for your research. If you are not sure, read the appropriate sections before making your selection.

☒ Life sciences ☐ Behavioural & social sciences ☐ Ecological, evolutionary & environmental sciences

For a reference copy of the document with all sections, see nature.com/documents/nr-reporting-summary-flat.pdf

# Life sciences study design

All studies must disclose on these points even when the disclosure is negative.

**Sample size**

This was an exploratory study to evaluate the safety and dose evaluation of B-VEC in DEB patients and hence no formal sample calculation was done. The pharmacology studies in diseased DEB animal models clearly demonstrated B-VEC was able to produce functional COL7 that is needed to treat the patient's underlying disease condition. The main objectives of the Phase 1/2 study in DEB patients were to evaluate safety, molecular correction and preliminary efficacy in 10 to 12 subjects. Results from completed study in this small number of patients clearly demonstrated safety, molecular correction and preliminary efficacy. Also, post hoc responder analysis of the clinical data from the Phase 1/2 study was sufficient for the calculation of an effect size that helped in determining the sample size for the pivotal Phase 3 study without the need for more additional patients in the Phase 1/2 study.

**Data exclusions**

Data from two subjects was excluded from the efficacy analysis. The pooled primary clinical efficacy data set consisted of wounds from 11 subjects. Three subjects rolled over from phase 2b to phase 2c and were then re counted as new subjects. The two patients were excluded in the responder analysis of the observed data because as per FDA guidance for efficacy wound healing had to be demonstrated at two consecutive time points weeks 8 and 10 or weeks 10 and 12. One of the patient dropped out of the study after 30 days after the initial dosing due to an inability to travel and had missing data that could not be imputed. The other subject was part of an exploratory evaluation for treatment of large chronic wound and was pre-established to be excluded from the responder analysis.

**Replication**

Three subjects rolled over from Phase 2a to 2b portion of the study however different wounds within the subjects were treated and evaluated except for one wound of a subject that did not close during the 2a phase was re-treated in phase 2b.
Replicates:
Figure 1
a–d: n=3 (wells for each condition);
e: Representative images of 9 injection sites (3 mice) of B-VEC and 3 injection sites of placebo (PBS);
f, g ,h bottom: Representative images of 3 injections (3 mice) of B-VEC with 2 injections (doses) each;
i: Representative images of 4 injection sites (4 mice) of B-VEC for each dose, high and low);
j: 4 areas on back treated per mouse (1 control and 3 B-VEC) and 4 mice total were tested;
k: Representative of 8 grafts treated with B-VEC (mice) and 2 placebo;
l: Representative of 8 grafts treated with B-VEC and 2 grafts treated with placebo.

**Randomization**

The study has a complete randomized-block design in which each subject serves as a block to receive all of the treatment conditions.

**Blinding**

The study was open label randomized study in which complete closure of the treated wounds were evaluated by the investigator at weeks 8, 10 and 12, In addition, complete wound closure evaluation at the 8, 10 and 12 week time points were conducted by two blinded evaluators. The blinded evaluation results showed similar trends in efficacy. For preclinical studies, investigators evaluating immunofluorescence microscopy and electron microscopy images were blinded. For in vitro western blot analysis experiments, investigators evaluating densitometry were blinded.

# Reporting for specific materials, systems and methods

We require information from authors about some types of materials, experimental systems and methods used in many studies. Here, indicate whether each material, system or method listed is relevant to your study. If you are not sure if a list item applies to your research, read the appropriate section before selecting a response.

## Materials & experimental systems

| n/a | Involved in the study | |
|---|---|---|
| ☐ | ☒ | Antibodies |
| ☒ | ☐ | Eukaryotic cell lines |
| ☒ | ☐ | Palaeontology and archaeology |
| ☐ | ☒ | Animals and other organisms |
| ☐ | ☒ | Human research participants |
| ☐ | ☒ | Clinical data |
| ☒ | ☐ | Dual use research of concern |

## Methods

| n/a | Involved in the study | |
|---|---|---|
| ☒ | ☐ | ChIP-seq |
| ☒ | ☐ | Flow cytometry |
| ☒ | ☐ | MRI-based neuroimaging |

# Antibodies

Antibodies used

C7 antibodies to the NCl domain, C7 antibodies to the NC2 domain.

**Table S5 WITH additional Ab and dilution column**

| Target molecule | Origin/isotype/type | Primary/ Secondary | source | CAT# | dilution |
|---|---|---|---|---|---|
| human type VII collagen | Rabbit, monoclonal, IgG | Primary | Sigma | HPA042420 | 1:250 |
| integrin alpha 6 (clone goH3) | Rat, IgG | Primary | BD Biosciences | 555734 | 1:500 |
| Anti-human type VII collagen NC1 domain antibody (clone NP185) | Mouse monoclonal IgG | Primary | Lab | Reference [1] | 1:100 |
| Anti-human type VII collagen NC2 domain antibody (clone LH24) | Mouse monoclonal IgM | Primary | Lab | Reference [2] | 1:5 |
| Anti-laminin 332 antisera (pKal) | Rabbit polyclonal IgG | Primary | Lab | Reference [3] | 1:300 |
| anti-Rabbit -AF 647 | Goat anti-Rabbit IgG | Secondary | Invitrogen | A21244 | 1:400 |
| Anti-Rat AF-594 | Goat anti-Rat IgG | Secondary | Invitrogen | A11007 | 1:400 |
| a-IgM488 Mouse IgM | Goat anti-Mouse IgM | Secondary | Invitrogen | A-21042 | 1:400 |
| anti-mouse IgG-AF 594 | Goat Anti Mouse | Secondary | Invitrogen | A11032 | 1:400 |
| Anti-Rb-594 | Goat anti-Rabbit I | Secondary | Invitrogen | A32740 | 1:400 |
| GAPDH | GAPDH(G-9) mouse monoclonal IgG | Primary | Santa Cruz | Sc365062, lot G2320 | 1:200 |

Validation

Antibodies were validated in previous publications or from manufacturer data sheets.

# Animals and other organisms

Policy information about studies involving animals; ARRIVE guidelines recommended for reporting animal research

Laboratory animals

Balb/C mice were obtained from The Jackson Laboratory. For xenografting, NOD/SCID mice were used (NOD.CB17-PrkdcSCID/J mice; stock 001303; The Jackson Laboratory).
Col7a1flNeo mice were established from a breeding pair donated by Dr. Leena Bruckner-Tuderman (Freiberg, Germany). Mice 6 to 8 weeks old were used. Animals were housed with the following conditions: 14 hour light/10 hour dark cycle, temperature of 18-23 degrees C, 40-60% humidity.

Wild animals

No wild animals were used.

Field-collected samples

No field-collected samples were used.

Ethics oversight

Stanford APLAC committee approved all animal studies.

Note that full information on the approval of the study protocol must also be provided in the manuscript.

# Human research participants

Policy information about studies involving human research participants

Population characteristics

DEB is a ultra rare genetic disease where the patients have a mutation in the COL7A1 gene and are unable to produce functional COL7 protein. The population selection for the study was based on confirmation of the genetic defect in the COL7A1 gene. 9 males and 3 females with generalized recessive dystrophic epidermolysis bullosa, ages ranging from 10-36 years, were included in the study.

Recruitment

Patients were recruited out of Dr. Marinkovich's Bullous Disease Clinic at Stanford University. Patients were recruited based on confirmation of a genetic mutation in the COL7A1 gene; there was no selection bias in recruiting or enrollment of patients in the Phase1/2 trial.

Ethics oversight

The study protocol, all its amendments, and the patient information sheet(s) were reviewed and approved by the appropriate independent Stanford Universities ethics committees. Written informed consent was obtained from patients or patients' legally authorized representatives. Patients received compensation to cover travel costs and meals for the day of the visit.

Note that full information on the approval of the study protocol must also be provided in the manuscript.

# Clinical data

Policy information about clinical studies

All manuscripts should comply with the ICMJE guidelines for publication of clinical research and a completed CONSORT checklist must be included with all submissions.

Clinical trial registration

(NCT03536143) May 24th 2018

| | |
|---|---|
| Study protocol | A Phase 1/11 Study of B-VEC, a Non-Integrating, Replication-Incompetent HSV Vector Expressing the Human Collagen VII Protein, for the Treatment of Dystrophic Epidermolysis Bullosa (DEB). |
| Data collection | Data was captured using case report forms and data was entered into an electronic data management system. Patients were recruited from Stanford Epidermolysis Bullosa clinic, starting from May 1, 2018 until Sept 1, 2019. Data collection was from May 3, 2018, until November 25, 2019. |
| Outcomes | Since the Phase1/2 trial of B-VEC was a first in man gene therapy trial, safety of B-VEC was the primary objective and was defined as the primary end point. B-VEC is a gene replacement corrective therapy hence evaluation of molecular correction and durability of wound closure associated with molecular correction were the secondary end points.<br>To evaluate safety and tolerability of repeat B-VEC use, adverse events (AEs), and changes in vitals, physical exam, and laboratory results, including anti-COL7 and anti-HSV-1 antibodies were assessed. Molecular correction was established by taking biopsies of the healed skin of the treated area and showing presence of functional COL7 protein by Immunofluorescence (IF) and formation of anchoring fibrils (AF) by immunoelectron microscopy (IEM). Wound healing was captured using a validated imaging device. |
