# Supplementary figures and images for: In vivo topical gene therapy for recessive dystrophic epidermolysis bullosa: a phase 1 and 2 trial
Source: Nat Med. 2022 Mar 28;28(4):780–8. doi: 10.1038/s41591-022-01737-y (PMC9018416; doi:10.1038/s41591-022-01737-y)

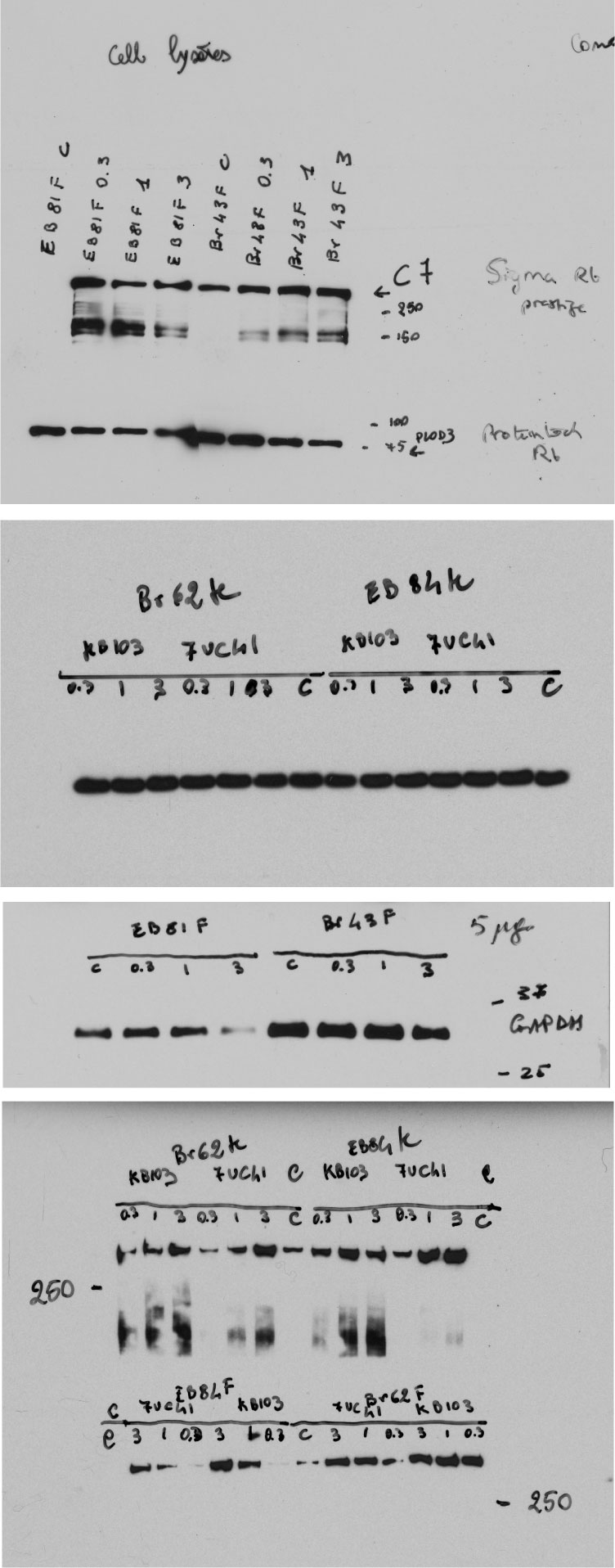

Supplement: Supplementary file 6 — Unprocessed Western blots [file 41591_2022_1737_MOESM6_ESM.jpg]
